# Health impact of seven herpesviruses on (pre)diabetes incidence and HbA_1c_: results from the KORA cohort

**DOI:** 10.1007/s00125-022-05704-7

**Published:** 2022-05-11

**Authors:** Tim Woelfle, Birgit Linkohr, Tim Waterboer, Barbara Thorand, Jochen Seissler, Marc Chadeau-Hyam, Annette Peters

**Affiliations:** 1 Institute of Medical Information Sciences, Biometry and Epidemiology, Ludwig-Maximilians University, Munich, Germany; 2 School of Public Health, Imperial College London, London, UK; 3 Institute of Epidemiology, Helmholtz Zentrum München, German Research Centre for Environmental Health, Neuherberg, Germany; 4 Division of Infections and Cancer Epidemiology, German Cancer Research Center (DKFZ), Heidelberg, Germany; 5 German Center for Diabetes Research (DZD), München-Neuherberg, Germany; 6 Diabetes Centre, Medical Clinic and Policlinic IV, University Hospital, Ludwig-Maximilians University, Munich, Germany

**Keywords:** Cytomegalovirus (CMV), Epstein–Barr virus (EBV), HbA_1c_, Herpes simplex virus 1 (HSV1), Herpes simplex virus 2 (HSV2), Human herpes virus 6 (HHV6), Human herpes virus 7 (HHV7), Incidence, Prediabetes, Varicella-zoster virus (VZV)

## Abstract

**Aims/hypothesis:**

The prevalence of type 2 diabetes is increasing worldwide, and previous studies have suggested that it is higher in individuals who are seropositive for herpesviruses. This study examines the prospective association of herpesviruses with (pre)diabetes to evaluate their potential role in diabetes aetiology.

**Methods:**

Two follow-up examinations of the German population-based KORA cohort (F4 and FF4) were used to identify participants with normal glucose tolerance at baseline, thus being at risk for (pre)diabetes (*n* = 1257). All participants had repeated OGTTs and antibody measurements for herpes simplex virus (HSV) 1 and 2, varicella-zoster virus, Epstein–Barr virus, cytomegalovirus (CMV) and human herpesvirus 6 and 7. Regression models were used to evaluate the association between serostatus with (pre)diabetes incidence after a 7 year follow-up and HbA_1c_.

**Results:**

HSV2 and CMV were associated with (pre)diabetes incidence after adjustment for sex, age, BMI, education, smoking, physical activity, parental diabetes, hypertension, lipid levels, insulin resistance and fasting glucose. Seropositivity of both viruses was also cross-sectionally associated with higher HbA_1c_ at baseline, with the association of HSV2 being independent of confounders, including the prevalence of (pre)diabetes itself. While seropositivity for multiple herpesviruses was associated with a higher incidence of (pre)diabetes, this association was not independent of confounders.

**Conclusions/interpretation:**

The associations of HSV2 and CMV serostatus with (pre)diabetes incidence indicate that these herpesviruses may contribute to the development of impaired glucose metabolism. Our results highlight the link between viral infection and (pre)diabetes, and the need for more research evaluating viral prevention strategies.

**Graphical abstract:**

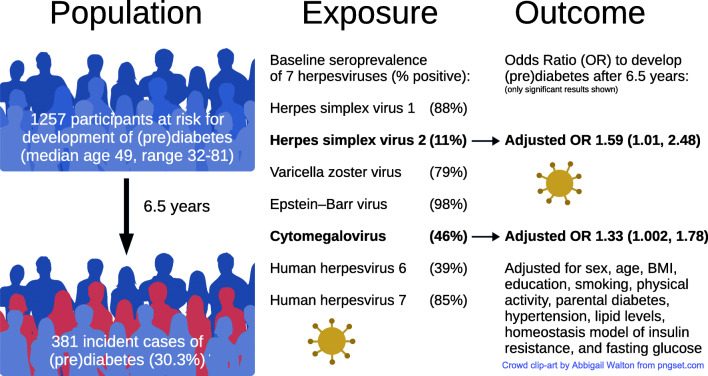

**Supplementary Information:**

The online version contains peer-reviewed but unedited supplementary material available at 10.1007/s00125-022-05704-7.

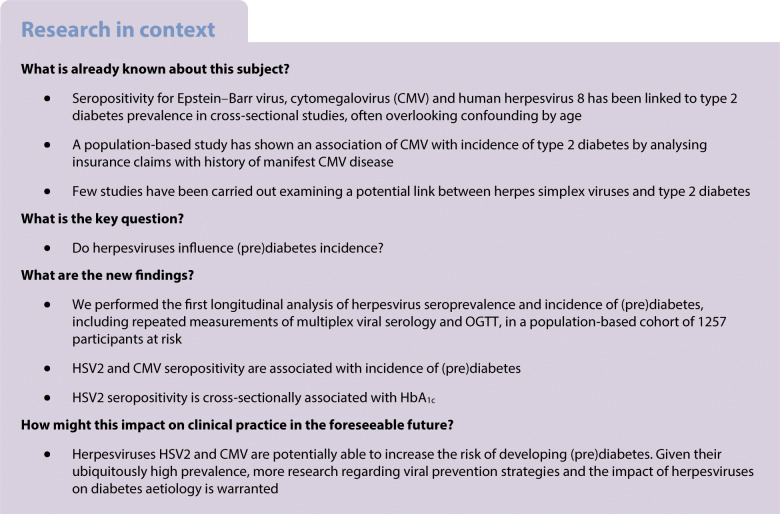



## Introduction

This work explores an intersection of the two major branches of epidemiology, communicable and non-communicable disease, examining the association of herpesviruses, arguably one of the most prevalent groups of viruses, with type 2 diabetes, arguably one of the most important metabolic diseases.

Eight herpesviruses are known to affect humans. In order of their scientific discovery, these are herpes simplex viruses (HSV) 1 and 2, varicella-zoster virus (VZV), Epstein–Barr virus (EBV), cytomegalovirus (CMV) and human herpesviruses (HHV) 6, 7 and 8. All cause lifelong latent infection in their hosts after usually mild systemic primary infections [1].

Type 2 diabetes is one of the most widespread metabolic diseases, with a 2019 worldwide prevalence estimate of 9.3%, exerting a high mortality burden mainly due to cardiovascular disease [2, 3]. Individuals may be diagnosed with prediabetes when they have impaired fasting glucose (IFG) or impaired glucose tolerance (IGT) [4]. Kowall et al have shown that the incidence rate of type 2 diabetes is much higher in people with prediabetes (up to 7.6% per person-year) compared with those with normal glucose tolerance (0.6% per person-year) [5].

Many behavioural and environmental risk factors for type 2 diabetes have been established, including unhealthy diet, obesity and inflammation [6–8]. Additionally, many genetic risk factors have been identified [9]. Until recently, an aetiological involvement of viruses in diabetes development has only been proposed for type 1 diabetes [10], with mostly enteroviruses and Coxsackie viruses having been suggested as potential risk factors [10, 11].

Type 2 diabetes and poor glycaemic control are associated with reduced function of the innate and adaptive immune system, and therefore type 2 diabetes increases the susceptibility to infections [12, 13]. For example, tuberculosis and type 2 diabetes have been shown to facilitate one another, creating a double burden of two epidemics in several countries [14]. Links between type 2 diabetes and viral infections have also been demonstrated, generally with the conclusion that diabetes precedes and increases the risk of the viral infection [15]. A recent meta-analysis estimated increased prevalence of severe acute respiratory syndrome coronavirus 2 (SARS-CoV-2; OR 10.8), hepatitis C (OR 3.6), hepatitis B (OR 1.6) and other viruses among type 2 diabetic patients [15].

With regard to human herpesviruses, an increased prevalence of HHV8 among type 2 diabetic patients has been reported in multiple populations [15–17]. Some cross-sectional studies have also demonstrated an association of HSV1 and CMV serostatus with prevalence of type 2 diabetes [15, 18–20], but the results were partly confounded by age and other demographic factors [21]. Moreover, the cross-sectional nature of these studies means that they cannot demonstrate chronology or causality [17]. Well-designed longitudinal studies are therefore required to elucidate the potential involvement of herpesvirus infection in the development of (pre)diabetes.

This study examines associations of the seven herpesviruses HSV1, HSV2, VZV, EBV, CMV, HHV6 and HHV7 with (pre)diabetes incidence in a longitudinal population-based cohort study. Additionally, associations with HbA_1c_ are examined cross-sectionally.

## Methods

### KORA study and participant selection

KORA (Cooperative Health Research in the Region of Augsburg) is a population-based health research platform in the south of Germany [22]. Our analyses are based on data from the F4 study (2006–2008) and the FF4 study (2013–2014), which are follow-up examinations of the KORA S4 health survey. For the present analysis, we defined F4 as our baseline sample and FF4 as the follow-up. The participants have undergone extensive phenotyping including viral multiplex serology for human herpesviruses as well as OGTT and HbA_1c_ at both F4 and FF4 [5]. All study methods were approved by the ethics committee of the Bavarian Chamber of Physicians, Munich (EC No. 06068).

Of the 3077 and 2279 participants originally included in the F4 and FF4 studies, we excluded those with inconclusive or missing viral serology, missing OGTT or a history of type 1 or drug-induced diabetes, leaving *n* = 2950 and *n* = 2129 participants at F4 and FF4, respectively. The overlap of *n* = 1967 participants was used for descriptive and cross-sectional analyses, with a median age of 54 years (range 32–81) at baseline; 968 (49.2%) participants were men. Incidence analysis was performed in *n* = 1257 participants at risk for (pre)diabetes (i.e. having normal glucose tolerance at baseline), with a median age of 49 years (range 32–81); 528 (42.0%) participants were men. Due to a technical failure in the assay for VZV antibodies in 427 samples, VZV analyses were performed in a subgroup of *n* = 1540 overlapping participants including *n* = 986 participants at risk for (pre)diabetes. Electronic supplementary material (ESM) Tables 1–3 provide a demographic overview, and ESM Fig. 1 shows a participant selection flowchart.

### (Pre)diabetes definition

Participants without prior diagnosis of type 2 diabetes had standard 75 g OGTT performed in the morning after overnight fasting. Diabetes status was defined according to glucose tolerance using the thresholds recommended by the American Diabetes Association [4]. Prediabetes was defined as IFG 5.6 mmol/l ≤ fasting glucose ≤6.9 mmol/l and/or IGT 7.8 mmol/l ≤2 h glucose ≤11.0 mmol/l; manifest type 2 diabetes was diagnosed if the upper cutoffs were exceeded in either of the two glucose measurements. The study uses a combined outcome of (pre)diabetes to maximise statistical power.

### Viral multiplex assays

Human herpesvirus serology was determined using multiplex serology, a suspension array-based immunoassay based on recombinant herpesvirus antigens bound to fluorescence-encoded microspheres [23]. Multiplex serology has been fully validated and has been used in numerous seroepidemiological studies [24, 25]. Antigen binding of serum antibodies was quantified through incubation with biotinylated goat α-human anti-IgM/IgG/IgA secondary antibodies and a reporter dye (streptavidin-R–phycoerythrin). Each bead set represents one antigen and consists of hundreds of beads whose median fluorescence intensities (MFI) are reported as the results [24]. Validation of this multiplex assay has already been performed successfully for herpesviruses 1–5, but is difficult for HHV6 and HHV7, which are still lacking gold standards [24, 25].

Patients were declared seropositive for a given virus if the MFI were above antigen-specific thresholds established previously (ESM Table 4) [24, 25]. HSV1, HSV2 and HHV7 were represented by a single antigen, VZV by two antigens, and EBV, CMV and HHV6 by four antigens. Patients were declared seropositive for VZV if either of the two antigens was above the MFI threshold; patients were declared seropositive for EBV, CMV, and HHV6 if at least two of the four antigens were above the MFI thresholds (ESM Table 4).

### Confounder variables

The following potential confounder variables were assessed at baseline and used for adjustment: sex, age, BMI, years of education (numerical), ever-smoking status (yes/no), leisure time physical activity (active/inactive [26]), parental diabetes (yes/no) and hypertension (yes/no, defined as >140/90 mmHg). In addition, baseline laboratory measures of total triacylglycerols (mmol/l), the ratio of total cholesterol to HDL-cholesterol (HDL-C), HOMA-IR and fasting serum glucose (mmol/l) were used as potential confounders [26]. HOMA-IR was calculated as previously described [26].

### Statistical analyses

Descriptive analyses were performed cross-sectionally (at baseline and follow-up) and longitudinally. This included quantification of seroprevalence for the various herpesviruses, as well as description of their co-occurrences and prevalence and incidence of (pre)diabetes.

Associations of herpesvirus serostatus at baseline (binary exposure variables) with (pre)diabetes incidence (binary outcome variable) were assessed using univariate and multivariate logistic regression models. We subsequently extended our models to include all seven viruses at once, which returned mutually adjusted effect size estimates for each separate virus. To account for the complex correlation and co-occurrence of the viruses, we also regressed the number of viruses that participants were seropositive for against (pre)diabetes incidence. All models were adjusted successively for the above-mentioned confounders.

We also adopted a variable selection approach using ‘least absolute shrinkage and selection operator’ (LASSO) penalised regression to identify a sparse set of viruses that jointly and complementarily associate with (pre)diabetes development [27]. Logistic LASSO models were calibrated using fivefold cross-validation, and the optimal penalty was defined as the one minimising the binomial deviance. To ensure our findings were not driven by outlying observations, the procedure was repeated on 1000 subsamples fitted on 80% of the full population, while keeping the proportion of incident (pre)diabetes cases and controls the same as in the full sample. Adopting a stability selection approach [28], the relevance of each separate predictor was evaluated by its selection proportion among the 1000 subsamples. Two versions of the LASSO regression models were run: one including serostatus data only, and one also including the same potential confounders as above.

To obtain the same sample size in the mutually adjusted and LASSO models as in most of the univariate models (*n* = 1257) despite antibody failure in the VZV F4 assays in 166/1257 participants, we used the VZV serostatus at FF4 as an approximation for baseline VZV serostatus for these participants in the mutually adjusted models. The suitability of this approach is backed by the high seroprevalence for VZV at both F4 (79%) and FF4 (83%), and the fact that infection with varicella usually occurs in childhood [29].

Missing values in confounding variables occurred for parental diabetes (482/1967, 24.5%), HOMA-IR (86/1967, 4.4%), fasting glucose (4/1967, 0.2%), years of education (4/1967, 0.2%), BMI (2/1967, 0.1%), ever-smoking status (1/1967, 0.05%), total triacylglycerols (1/1967, 0.05%) and total cholesterol/HDL-C (1/1967, 0.05%). These were imputed from the other confounding variables using linear or logistic regression models.

Associations of herpesvirus serostatus with HbA_1c_ (continuous variable) were assessed cross-sectionally using linear regression in both univariate and multivariate models adjusting for the confounders stated above except fasting glucose. Additionally, models further including prevalent (pre)diabetes coded as two binary variables (prediabetes and type 2 diabetes), and models including all viruses at once to estimate mutually adjusted effects, were examined.

We report point estimates and 95% CI of the effect size estimates. Regression and χ^2^ *p* values are based on two-sided tests and the level for statistical significance was defined as *p*≤0.05. Statistical analyses were performed using the statistical software language R, version 3.6.1.

## Results

### (Pre)diabetes and herpesvirus prevalence and incidence

The prevalence of prediabetes (IFG/IGT) was 27.5% at F4 and 36.2% at FF4, while that of type 2 diabetes was 8.5% at F4 and 14.6% at FF4 (ESM Table 1). Among the 1257 participants with normal glucose tolerance at baseline, 364 developed prediabetes and 17 developed type 2 diabetes over a mean follow-up duration of 6.5 years (ESM Fig. 2). Age, BMI, smoking and education were associated with both prediabetes and type 2 diabetes (ESM Fig. 3).

EBV was the most prevalent herpesvirus at F4 (98%), followed by HSV1 (88%), HHV7 (85%), VZV (79%), CMV (46%), HHV6 (39%) and HSV2 (11%) (see ESM Fig. 4). The mean number of herpesviruses that participants were seropositive for was 4.4 ± 1.1 at F4 and 4.7 ± 1.1 at FF4 in the 1540 overlapping participants with complete serology (CI for difference: 0.21, 0.37). A third of these were positive for more viruses at FF4 than at F4 (34%), 54% were positive for the same number of viruses, and only 12% were positive for fewer viruses (ESM Fig. 5). Most participants with seroconversions in either direction had antibody reactivities close to threshold at F4 (see ESM Figs 6–22).

### Associations of herpesvirus seroprevalence with (pre)diabetes incidence

**Fig. 1:**
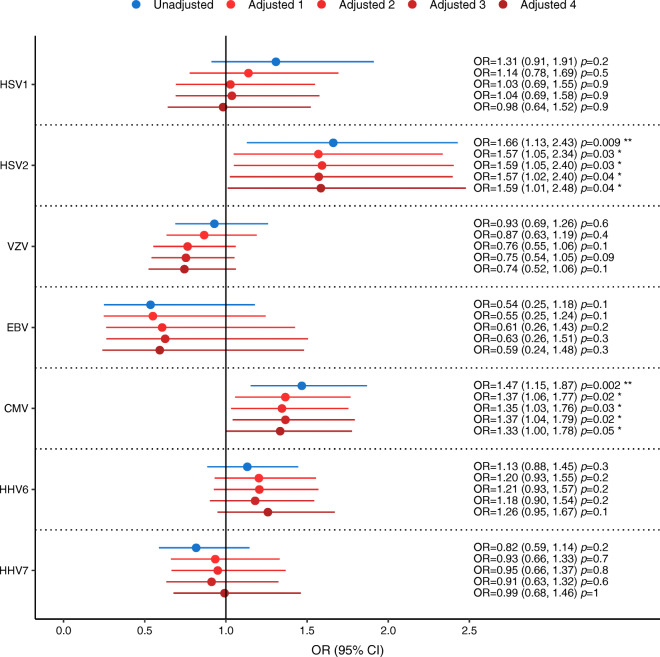
Associations of herpesvirus seropositivity with incidence of (pre)diabetes using the serostatus of each virus separately as the predictor in logistic regression models (*n* = 1257, except for VZV for which *n* = 986; 95% CI in brackets). Results are presented for unadjusted models and for models adjusted for: (1) sex and age, (2) adjusted 1 plus BMI, education, smoking and physical activity, (3) adjusted 2 plus parental diabetes, hypertension, triacylglycerols, total cholesterol/HDL-C and HOMA-IR, and (4) adjusted 3 plus fasting glucose. *p≤0.05, **p≤0.01

Of the seven herpesviruses examined, HSV2 and CMV were associated with (pre)diabetes incidence among the 1257 participants with normal glucose tolerance at baseline. These associations were independent of sex, age, BMI, smoking, education, physical activity, parental diabetes, hypertension, lipid levels, insulin resistance and fasting glucose (Fig. 1, ESM Tables 5–11 and ESM Figs 23–29).

Participants who were seropositive for HSV2 had 66% higher crude odds of developing (pre)diabetes during the 6.5 years between F4 and FF4 compared with those who were seronegative (OR 1.66, CI 1.13, 2.43). There was a slight effect attenuation when adjusting for age, hypertension and triacylglycerols, but not when adjusting for the other confounders (ESM Fig. 24, ESM Table 6). The adjusted model including all confounders indicated an independent association of HSV2 with (pre)diabetes incidence (OR 1.59, CI 1.01, 2.48) (Fig. 1).

We also identified an association of CMV and the incidence of (pre)diabetes (OR 1.47, CI 1.15, 1.87), which was partially explained by age, hypertension and triacylglycerols, but not by the other potential confounders. The adjusted OR of 1.33 (CI 1.00, 1.78) demonstrated an independent association of CMV with (pre)diabetes incidence (ESM Fig. 27, ESM Table 9). Including all viruses in a single mutually adjusted model yielded very similar results, and identified HSV2 and CMV as jointly contributing to (pre)diabetes incidence (ESM Fig. 30).

**Fig. 2:**
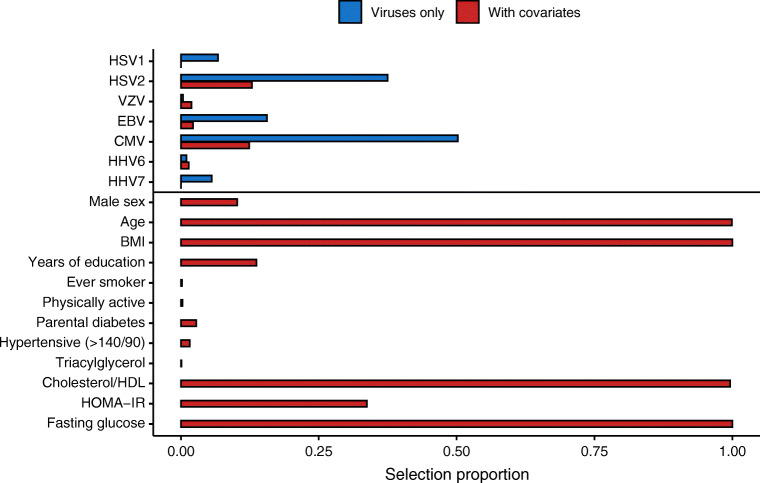
Selection proportion of viruses and confounders in two logistic LASSO models on (pre)diabetes incidence (*n* = 1257 participants). The first model (blue) only includes the serostatus for the seven assayed herpesviruses, and the second model (red) further includes confounders. We report the selection proportion calculated over 1000 calibrated models fitted on 80% of the full population, each including the same proportion of incident cases. For each model, the penalty was calibrated using fivefold cross-validation. The selection proportion of each variable was derived by summing the number of times it was included across the 1000 models β (95% CI)

LASSO stability analysis showed that HSV2 (selection proportion 37.5%) and CMV (selection proportion 50.2%) were by far the most stably selected viruses (Fig. 2). When including all confounders in the model, age, BMI, total cholesterol/HDL-C and fasting glucose were systematically included in the models (selection proportions 100%), and the selection proportions for viral serostatus decreased. Nevertheless, both HSV2 and CMV remained the most frequently selected viruses (selection proportions 12.9% and 12.4%, respectively).

### Cross-sectional association of seropositivity with baseline HbA_1c_

**Fig. 3:**
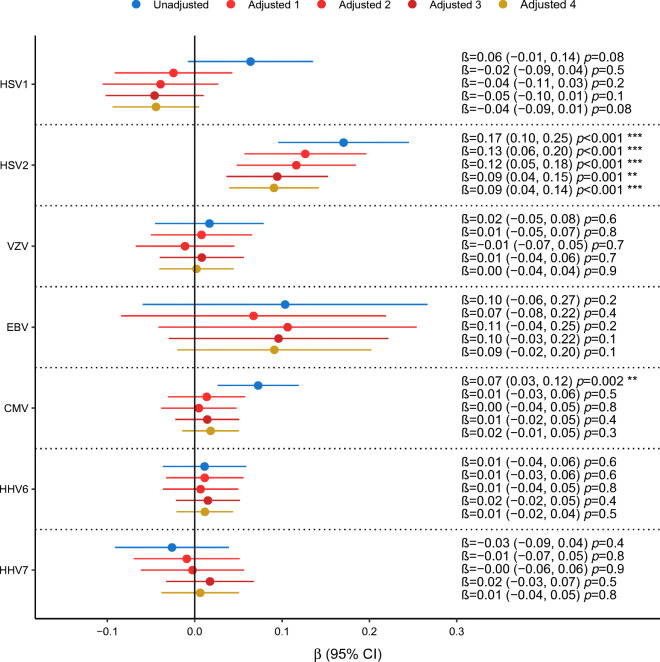
Association of each herpesvirus serostatus separately with HbA_1c_. Results are reported for the serostatus and HbA_1c_ measured at baseline (*n* = 1967 participants, except for VZV for which *n* = 1540). We report the regression coefficients (β and 95% CI) for unadjusted models and models adjusted for: (1) sex and age, (2) adjusted 1 plus BMI, education, smoking and physical activity, (3) adjusted 2 plus parental diabetes, hypertension, triacylglycerols, total cholesterol/HDL-C and HOMA-IR, and (4) adjusted 3 plus prevalence of (pre)diabetes. **p≤0.01, ***p≤0.001

HSV2 and CMV seropositivity was significantly associated cross-sectionally with baseline HbA_1c_, indicating long-term hyperglycaemia, with crude β estimates of 0.17 (CI 0.10, 0.25) and 0.07 (CI 0.03, 0.12), respectively. None of the other viruses were significantly associated with HbA_1c_ (Fig. 3).

The association of HSV2 with HbA_1c_ survived adjustment for confounders (adjusted effect size estimate 0.09; CI 0.04, 0.14). Interestingly, it even survived adjustment for prediabetes and type 2 diabetes prevalence (Fig. 3). Results from mutually adjusted models including all viruses together yielded highly comparable associations of HSV2 and CMV with HbA_1c_ (ESM Fig. 31).

### Association of combinations of multiple herpesviruses with (pre)diabetes incidence

**Fig. 4:**
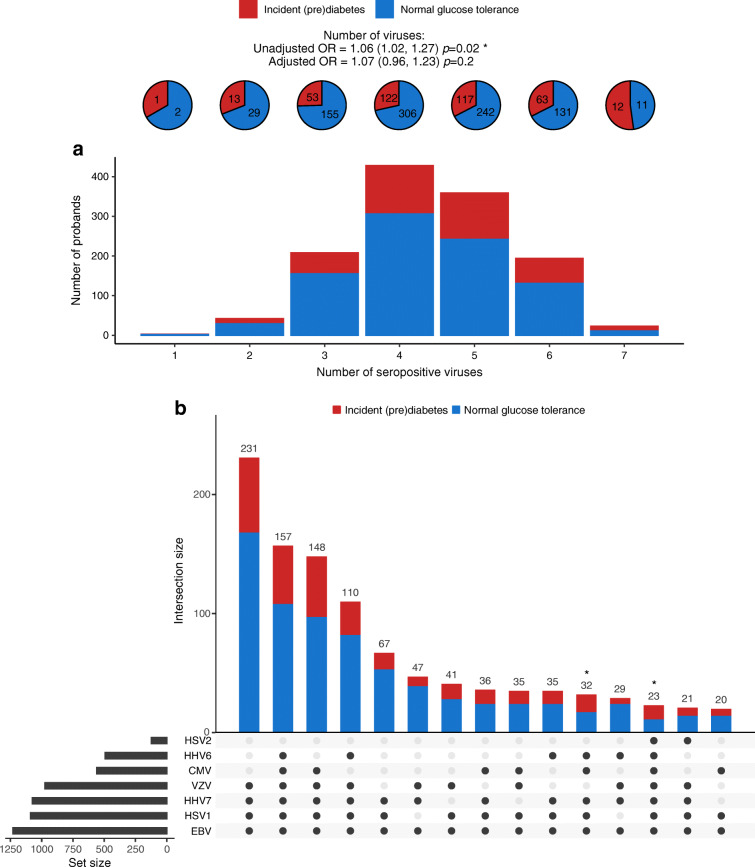
(**a**) Number of viruses that participants were seropositive for at baseline in relation to (pre)diabetes incidence (*n* = 1257). (**b**) Baseline viral co-occurrence with the 15 most common combinatorial patterns representing 1032 of 1257 at-risk participants in bars coloured by (pre)diabetes incidence; *p*=0.072 by χ^2^ test. Combinations with fewer than 20 participants each are not shown and account for the remaining 225 participants. *p≤0.05

The association of the number of viruses that participants were seropositive for with incidence of (pre)diabetes in at-risk participants (i.e. normal glucose tolerance at F4) suggested an increase in the odds of (pre)diabetes of 1.06 (CI 1.02, 1.27) per virus at baseline (Fig. 4a), resulting in an OR of 1.50 for someone carrying all seven herpesviruses. However, this association did not survive adjustment for confounders.

Looking at co-occurrence patterns of viral seropositivity, there did not seem to be specific combinations of viruses that were associated with (pre)diabetes more than others, as indicated by the *p* value 0.072 for the χ^2^ test for the 15 most common co-occurrence patterns at baseline (Fig. 4b). However, two combinations stood out when examining the standardised residuals: the combination seropositive for all viruses except VZV and HSV2 (32 participants) had a standardised residual of 2.2 (nominal *p*=0.03), and the combination seropositive for all seven viruses (23 participants) had a standardised residual of 2.4 (nominal *p*=0.02), indicating higher than average proportions of incident (pre)diabetes.

## Discussion

As far as we are aware, this study is the first to examine the association of the seroprevalence of seven herpesviruses with incidence of (pre)diabetes in a population-based longitudinal cohort, utilising repeated OGTT measurements (the diabetes diagnosis gold standard) and multiplex viral serology. By limiting our incidence analysis to participants with normal glucose tolerance at baseline, we reduced the risk of reverse causality.

We found an association of seropositivity for HSV2 and CMV with (pre)diabetes incidence. Multivariate analyses suggested that these two viruses consistently and complementarily contributed to (pre)diabetes incidence independently of sex, age, BMI, education, smoking, physical activity, parental diabetes, hypertension, lipid levels, insulin resistance and fasting glucose. Our variable selection approach suggested that, while (pre)diabetes incidence was primarily explained by age, BMI, cholesterol and fasting glucose, both HSV2 and CMV added additional complementary risk information, despite high viral prevalence and co-occurrence.

Moreover, HSV2 was cross-sectionally associated with HbA_1c_ independently of the confounders described above and even the prevalence of (pre)diabetes itself. Although it is unlikely that slightly increased blood glucose levels in the non-diabetic range compromised the immune system, cross-sectional modelling cannot distinguish causal effect directions.

The pathomechanisms for the potential involvement of HSV2 and CMV in (pre)diabetes development remain to be elucidated. Both viruses cause chronic infections and potentially modulate the immune system [30], which in turn influences the endocrine system. It has been established that there are other as yet unknown causes of type 2 diabetes development besides the metabolic syndrome [31].

While herpesviruses are persistent in their hosts, they may not always be detected by antibodies in blood due to changes in either the host immune system or viral activity. Infection with most herpesviruses usually occurs in early childhood, much earlier than at the median age at recruitment (54 years), but infections at an older age are possible. The seroconversions observed thus may represent incident cases, but are more likely to be due to an increased antibody reactivity of a previously undetectable virus. Similarly, a person who loses seropositivity cannot be considered healed of the virus but is much more likely to be in an undetectable latency state. These interpretations are supported by the fact that participants with seroconversions in either direction had MFI levels closer to the threshold at baseline than others.

### Comparison with previous evidence

A Korean study by Yoo et al published in 2019 linked a history of manifest CMV disease as evidenced by insurance claims to incidence of type 2 diabetes [32]. It reported an adjusted OR of 2.60 (CI 1.68, 3.95), which is quite a bit larger than our adjusted OR of 1.33 (CI 1.00, 1.78). This difference may be explained by the fact that Yoo et al were considering history of manifest CMV disease rather than CMV serostatus, leading to only 576 adult cases in a database encompassing the entire South Korean population of 50 million. They explain that manifest CMV disease has a higher impact on the overall immune system and inflammatory state than subclinical CMV infection [32]. Serostatus captures both manifest CMV disease (very rare) and subclinical infection, making our results more relevant for a much larger proportion of the population. CMV has also been found histopathologically in the islets of Langerhans in the pancreas in type 2 diabetes patients but not in controls, further increasing the plausibility of a causal role of CMV in the development of type 2 diabetes [33].

With regard to the other herpesviruses examined in this study, no association as clear as that with CMV has been described in the literature. Of note, the incidence of herpes zoster appears to be increased in type 2 diabetes patients [34]. A recent study found a significantly increased prevalence of EBV in type 2 diabetes patients, but not of CMV and HSV1 [35]. Haeseker et al found an association of type 2 diabetes with high IgG titres of HHV6 and EBV but not of CMV [30]. Piras et al examined differences in the viral DNA counts as well as antibody titres for EBV, CMV, HHV6, HHV7 and HHV8, and found differences between diabetic patients and controls only for HHV8 [36]. These diverse and sometimes contradictory results show that cross-sectional designs are not optimal for exploration of the links between viral infection and type 2 diabetes, due to the long latency of both the infection and the subclinical stages of diabetic conditions. Moreover, many demographic confounders as well as nuanced differences in populations can affect the results.

We could not find any studies examining the relation of HSV2 and (pre)diabetes in a general population, let alone showing a significant increase in (pre)diabetes incidence among seropositive individuals or an association with HbA_1c_ independent of typical demographic factors, like our study did. One paper showed no association of HSV2 with IFG/IGT, but this study was based on a group of HIV patients receiving antiretroviral therapy, thus hampering generalisability [37]. HSV2 may be the least prevalent of the herpesviruses, but it has nonetheless been shown to infect one in ten people globally [38]. Because of the large worldwide burden of herpesviruses, vaccines are already in development, an endeavour that should be intensified both academically as well as financially [39].

### Limitations and outlook

Serology does not fully capture past infections. Viral antibody concentrations are influenced by the severity and strength of the immune reaction upon primary infection, the state of the immune system, virus–host interaction and potential recurrent infections, among others. A certain instability of serostatus between the two timepoints was observed (ESM Figs 6–22), which cannot simply be explained by incident cases. We do not have any information on acute herpesvirus manifestations in the KORA study (e.g. prevalence and frequency of orolabial and genital herpes or zoster disease, history of varicella or infectious mononucleosis, etc.), making it hard to determine the potential reasons for the observed seroconversions.

Another limitation is the lack of validation of the viral multiplex assay for HHV6 and HHV7, mainly because no universally agreed upon gold standards exist, as discussed by Brenner et al, who developed the multiplex assay used in the KORA study [24]. Even though this may limit the possibility of comparing prevalence and incidence levels across studies, we believe that the relative intra-study levels are meaningful for risk assessment and association.

Finally, the medium sample size and the loss of follow-up between F4 and FF4 limit the statistical power of this study, even though approximately two-thirds of the 3077 participants at F4 participated at FF4 as well. Comparing the 1967 included participants with the 1100 non-participants shows that the non-participants were on average older and less healthy at baseline, with a much higher proportion of prevalent type 2 diabetes (8.5% in participants vs 15.7% in non-participants) and higher proportions of prevalent HSV1 and CMV (ESM Table 2). However, when comparing the 1257 at-risk participants with the 535 at-risk non-participants, these differences decrease (ESM Table 3). Nonetheless, we cannot fully exclude the possibility of healthy volunteer bias.

Using LASSO methods, HSV2 and CMV were selected as jointly explaining incident (pre)diabetes in complement of established risk factors. However, the selection proportions using stability LASSO methods remained rather low. This may be indicative of heterogeneity in the study population, which suggests that there are potentially subgroups of patients in whom the herpesviruses are particularly relevant. Therefore, larger population cohorts such as the UK Biobank or the German National Cohort may be valuable resources to confirm and extend the findings from this study [25].

### Conclusion

This study found novel prospective associations of HSV2 and CMV seropositivity with incidence of (pre)diabetes after adjustment for sex, age, BMI, education, smoking, physical activity, parental diabetes, hypertension, lipid levels, insulin resistance and fasting glucose. For HSV2, our findings are further strengthened by the cross-sectional association of serostatus with HbA_1c_, independent of confounders and even of the prevalence of (pre)diabetes itself. These results highlight the link between viruses and (pre)diabetes, and the need for more research evaluating public health viral prevention strategies, possibly including the development of effective vaccines against herpesviruses.

## Supplementary information

ESM 1 (PDF 1980 kb)

## Data Availability

The informed consent given by KORA study participants does not cover data posting in public databases. However, data are available upon request by means of a project agreement (https://epi.helmholtz-muenchen.de) subject to approval by the KORA Board.

## Abbreviations

CMV: Cytomegalovirus
EBV: Epstein–Barr virus
HDL-C: HDL-cholesterol
HHV: Human herpesvirus
HSV: Herpes simplex virus
IFG: Impaired fasting glucose
IGT: Impaired glucose tolerance
KORA: Cooperative Health Research in the Region of Augsburg
LASSO: Least absolute shrinkage and selection operator
MFI: Median fluorescence intensity
VZV: Varicella-zoster virus
