# Supplementary material for: Health impact of seven herpesviruses on (pre)diabetes incidence and HbA1c: results from the KORA cohort
Source: Diabetologia. 2022 May 11;65(8):1328–38. doi: 10.1007/s00125-022-05704-7 (PMC9090457; doi:10.1007/s00125-022-05704-7)
Supplement: Supplementary file 1 — (PDF 1980 kb) [file 125_2022_5704_MOESM1_ESM.pdf]

**Health impact of seven herpesviruses on (pre)diabetes incidence and HbA1c: results from the KORA cohort**

## Electronic supplementary material – contents


ESM Table 1: Demographical overview at baseline (F4) and follow–up (FF4) ... 3

ESM Table 2: Comparison of baseline (F4) characteristics of participants and non–participants ... 3

ESM Table 3: Comparison of baseline (F4) characteristics of at risk participants and at risk non–participants ... 4

ESM Table 4: Antigens for 7 human herpesviruses ... 4

ESM Table 5: Individual model coefficients for incidence models for HSV1 ... 5

ESM Table 6: Individual model coefficients for incidence models for HSV2 ... 5

ESM Table 7: Individual model coefficients for incidence models for VZV ... 6

ESM Table 8: Individual model coefficients for incidence models for EBV ... 6

ESM Table 9: Individual model coefficients for incidence models for CMV ... 7

ESM Table 10: Individual model coefficients for incidence models for HHV6 ... 7

ESM Table 11: Individual model coefficients for incidence models for HHV7 ... 8

ESM Figure 1: Flowchart of participant selection ... 9

ESM Figure 2: Comparing glucose tolerance status at baseline (F4) and follow–up (FF4) with on average 6.5 years in between (n=1967) ... 9

ESM Figure 3: T2D risk factors age, overweight, smoking, and poor–education at F4 timepoint ... 10

ESM Figure 4: Viral seroprevalences at baseline (F4) and follow–up (FF4) for the seven herpesviruses under investigation (n=1967, except for VZV n=1540) ... 10

ESM Figure 5: Changes of number of seropositive viruses per person from F4 to FF4 (n=1540) (B). ... 11

ESM Figure 6: Overview of serostatus change for HSV1 gG antigen ... 11

ESM Figure 7: Overview of serostatus change for HSV2 mgG unique antigen ... 12

ESM Figure 8: Overview of serostatus change for VZV gI(ORF67) antigen ... 12

ESM Figure 9: Overview of serostatus change for VZV gE(ORF68) antigen ... 13

ESM Figure 10: Overview of serostatus change for EBV Zebra antigen ... 13

ESM Figure 11: Overview of serostatus change for EBV EBNA–1 antigen ... 14

ESM Figure 12: Overview of serostatus change for EBV EA–D antigen ... 14

ESM Figure 13: Overview of serostatus change for EBV VCA p18 antigen ........ 15
ESM Figure 14: Overview of serostatus change for CMV pp28 antigen ........ 15
ESM Figure 15: Overview of serostatus change for CMV pp52 antigen ........ 16
ESM Figure 16: Overview of serostatus change for CMV pp65 antigen ........ 16
ESM Figure 17: Overview of serostatus change for CMV pp150 Nter antigen ........ 17
ESM Figure 18: Overview of serostatus change for HHV6 IE1A trunc. antigen ........ 17
ESM Figure 19: Overview of serostatus change for HHV6 p100 trunc. antigen ........ 18
ESM Figure 20: Overview of serostatus change for HHV6 IE1B trunc. antigen ........ 18
ESM Figure 21: Overview of serostatus change for HHV6 p101K trunc. antigen ........ 19
ESM Figure 22: Overview of serostatus change for HHV7 U14 antigen ........ 19
ESM Figure 23: Contingency table and contribution of individual covariates for the association of HSV1 with (pre)diabetes incidence ........ 20
ESM Figure 24: Contingency table and contribution of individual covariates for the association of HSV2 with (pre)diabetes incidence ........ 20
ESM Figure 25: Contingency table and contribution of individual covariates for the association of VZV with (pre)diabetes incidence ........ 20
ESM Figure 26: Contingency table and contribution of individual covariates for the association of EBV with (pre)diabetes incidence ........ 21
ESM Figure 27: Contingency table and contribution of individual covariates for the association of CMV with (pre)diabetes incidence ........ 21
ESM Figure 28: Contingency table and contribution of individual covariates for the association of HHV6 with (pre)diabetes incidence ........ 21
ESM Figure 29: Contingency table and contribution of individual covariates for the association of HHV7 with (pre)diabetes incidence ........ 22
ESM Figure 30: Mutually adjusted associations of herpesvirus seropositivity with incidence of (pre)diabetes (n=1257, 95% CI in brackets) ........ 23
ESM Figure 31: Mutually adjusted cross–sectional association of herpesvirus serostatus with HbA1c at baseline (n=1967, 95% CI in brackets) ........ 24

## ESM Tables

*ESM Table 1: Demographical overview at baseline (F4) and follow–up (FF4)*

| | **Main analyses (*n*=1967)** | | **VZV (due to antibody failure *n*=1540)** | |
|---|---|---|---|---|
| | **Baseline (F4)** | **Follow–up (FF4)** | **Baseline (F4)** | **Follow–up (FF4)** |
| **Male** | 968 (49.2%) | | 765 (49.7%) | |
| **Median age (years)** | 54 (range 32–81) | 61 (range 39–88) | 54 (range 32–81) | 61 (range 39–88) |
| **Median BMI (kg/m²)** | 27 (IQR 24–30) | 27 (IQR 24–31) | 27 (IQR 24–30) | 27 (IQR 24–31) |
| **Median years of education** | 11 (IQR 10–13) | 11 (IQR 10–13) | 11 (IQR 10–13) | 11 (IQR 10–13) |
| **Percent ever smoker (median packyears)** | 57.9% (15.0 PY) | 58.1% (15.4 PY) | 58.0% (15.0 PY) | 58.1% (15.3 PY) |
| **Number IFG/IGT** | 542 (27.5%) | 713 (36.2%) | 422 (27.4%) | 561 (36.4%) |
| **Number T2D** | 168 (8.5%) | 287 (14.6%) | 132 (8.6%) | 233 (15.1%) |

*ESM Table 2: Comparison of baseline (F4) characteristics of participants and non–participants*

| | **Participants (*n*=1967)** | **Non–participants (*n*=1110)** | **Difference p–value** |
|---|---|---|---|
| **Male** | 968 (49.2%) | 517 (46.6%) | =0.2 |
| **Median age (years)** | 54 (IQR 43-64, range 32-81) | 62 (IQR 47-72, range 32-81) | **<0.001** |
| **Median BMI (kg/m²)** | 26.68 (IQR 24.16-29.91, range 16.32-51.65), 2 (0.1%) NA | 27.53 (IQR 24.655-30.82, range 16.05-55.99), 15 (1.4%) NA | **<0.001** |
| **Median years of education** | 11 (IQR 10-13, range 8-17), 4 (0.2%) NA | 10 (IQR 10-13, range 8-17), 1 (0.1%) NA | **<0.001** |
| **Ever smoker** | 1139 (57.9%), 1 (0.1%) NA | 655 (59.3%), 5 (0.5%) NA | =0.5 |
| **Physically active** | 1150 (58.5%) | 521 (47.1%), 5 (0.5%) NA | **<0.001** |
| **Parental diabetes** | 451 (30.4%), 482 (24.5%) NA | 190 (29.7%), 470 (42.3%) NA | =0.8 |
| **Hypertension** | 328 (16.7%), 3 (0.2%) NA | 221 (20.0%), 5 (0.5%) NA | **=0.03** |
| **Median trigly-cerides (mmol/l)** | 1.17 (IQR 0.79-1.66, range 0.24-10.25), 1 (0.1%) NA | 1.23 (IQR 0.87-1.78, range 0.25-18.59), 5 (0.5%) NA | **<0.001** |
| **Median total cholesterol / HDL–C** | 3.91 (IQR 3.21-4.73, range 1.87-11.71), 1 (0.1%) NA | 3.91 (IQR 3.26-4.77, range 1.84-9.4), 5 (0.5%) NA | =0.2 |
| **Median HOMA–IR** | 1.95 (IQR 1.35-2.93, range 0.3-26.03), 86 (4.4%) NA | 2.12 (IQR 1.47-3.29, range 0.56-24.82), 153 (13.8%) NA | **<0.001** |
| **Median fasting glucose (mmol/l)** | 5.17 (IQR 4.89-5.61, range 3.61-14), 4 (0.2%) NA | 5.28 (IQR 4.89-5.83, range 3.61-18.94), 35 (3.2%) NA | **<0.001** |
| **HSV1 serostatus** | 1731 (88.0%) | 969 (90.8%), 43 (3.9%) NA | **=0.02** |
| **HSV2 serostatus** | 211 (10.7%) | 133 (12.5%), 43 (3.9%) NA | =0.2 |
| **VZV serostatus** | 1345 (78.5%), 253 (12.9%) NA | 684 (78.4%), 238 (21.4%) NA | =1 |
| **EBV serostatus** | 1926 (97.9%) | 1047 (98.1%), 43 (3.9%) NA | =0.8 |
| **CMV serostatus** | 903 (45.9%) | 574 (53.8%), 43 (3.9%) NA | **<0.001** |
| **HHV6 serostatus** | 768 (39.0%) | 427 (40.0%), 43 (3.9%) NA | =0.6 |
| **HHV7 serostatus** | 1672 (85.0%) | 906 (84.9%), 43 (3.9%) NA | =1 |
| **Number IFG/IGT** | 542 (27.6%) | 311 (28.0%) | =0.8 |
| **Number T2D** | 168 (8.5%) | 174 (15.7%) | **<0.001** |
| **Number T1D / drug–induced diabetes** | 0 | 9 (0.8%) | **<0.001** |
| **Number missing oGTT** | 0 | 81 (7.3%) | **<0.001** |

*ESM Table 3: Comparison of baseline (F4) characteristics of at risk participants and at risk non–participants*

| | **At risk participants (*n*=1257)** | **At risk non–participants (*n*=535)** | **Difference p–value** |
|---|---|---|---|
| **Male** | 528 (42.0%) | 216 (40.4%) | =0.6 |
| **Median age (years)** | 49 (IQR 41-59, range 32-81) | 53 (IQR 42-66.5, range 32-81) | **<0.001** |
| **Median BMI (kg/m$^2$)** | 25.595 (IQR 23.32-28.4775, range 16.32-46.73), 1 (0.1%) NA | 25.815 (IQR 23.265-29.02, range 17.5-44.79), 1 (0.2%) NA | =0.5 |
| **Median years of education** | 11 (IQR 10-13, range 8-17), 4 (0.3%) NA | 11 (IQR 10-13, range 8-17) | **=0.02** |
| **Ever smoker** | 728 (58.0%), 1 (0.1%) NA | 316 (59.4%), 3 (0.6%) NA | =0.6 |
| **Physically active** | 769 (61.2%) | 267 (50.2%), 3 (0.6%) NA | **<0.001** |
| **Parental diabetes** | 266 (26.2%), 242 (19.3%) NA | 96 (26.6%), 174 (32.5%) NA | =0.9 |
| **Hypertension** | 131 (10.4%), 2 (0.2%) NA | 75 (14.1%), 3 (0.6%) NA | **=0.03** |
| **Median trigly-cerides (mmol/l)** | 1.01 (IQR 0.71-1.44, range 0.24-6.93) | 1.09 (IQR 0.78-1.59, range 0.26-6.33) | **=0.003** |
| **Median total cholesterol / HDL–C** | 3.67 (IQR 3.02-4.43, range 1.87-8.26) | 3.69 (IQR 3.12-4.555, range 1.84-7.94) | =0.2 |
| **Median HOMA–IR** | 1.67 (IQR 1.19-2.25, range 0.3-11.72), 8 (0.6%) NA | 1.73 (IQR 1.19-2.3675, range 0.56-15.11), 13 (2.4%) NA | =0.1 |
| **Median fasting glucose (mmol/l)** | 5 (IQR 4.72-5.22, range 3.61-5.5) | 5 (IQR 4.72-5.22, range 3.89-5.5) | =0.4 |
| **HSV1 serostatus** | 1089 (86.6%) | 453 (88.6%), 24 (4.5%) NA | =0.3 |
| **HSV2 serostatus** | 123 (9.8%) | 78 (15.3%), 24 (4.5%) NA | **=0.001** |
| **VZV serostatus** | 840 (77.0%), 166 (13.2%) NA | 333 (79.9%), 118 (22.1%) NA | =0.3 |
| **EBV serostatus** | 1230 (97.9%) | 499 (97.7%), 24 (4.5%) NA | =0.9 |
| **CMV serostatus** | 560 (44.6%) | 250 (48.9%), 24 (4.5%) NA | =0.1 |
| **HHV6 serostatus** | 492 (39.1%) | 218 (42.7%), 24 (4.5%) NA | =0.2 |
| **HHV7 serostatus** | 1075 (85.5%) | 443 (86.7%), 24 (4.5%) NA | =0.6 |

*ESM Table 4: Antigens for 7 human herpesviruses*

| **Virus** | **Antigen** | **Description** | **MFI–Threshold** |
|---|---|---|---|
| **HSV1** | gG | Glyoprotein G | 100 |
| **HSV2** | mgG unique | Unique sequence of membrane–bound part of mature glycoprotein G | 100 |
| **VZV (1 / 2)** | gI(ORF67)<br>gE(ORF68) | Glycoprotein I<br>Glycoprotein E | 100<br>80 |
| **EBV (2 / 4)** | Zebra<br>EBNA–1<br>EA–D<br>VCA p18 | Virus protein ZEBRA<br>Nuclear antigen 1 (EBNA–1)<br>Early antigen–diffuse (EA–D)<br>Viral capsid antigen (VCA p18) | 150<br>150<br>150<br>200 |
| **CMV (2 / 4)** | pp 28<br>pp 52<br>pp 65<br>pp150 Nter | Protein pp 28<br>Protein pp 52<br>Protein pp 65<br>Protein pp 150 (N–terminus) | 150<br>150<br>150<br>150 |
| **HHV6 (2 / 4)** | IE1A trunc.<br>p100 trunc.<br>IE1B trunc.<br>p101K trunc. | 6A Immediate–Early 1 Protein<br>6A antigenic tegument protein<br>6B Immediate–Early 1 Protein variant B<br>6B antigenic tegument protein | 50<br>50<br>50<br>50 |
| **HHV7** | U14 | Protein U14, putative structural function | 100 |

*ESM Table 5: Individual model coefficients for incidence models for HSV1*

| | Adjusted model 1 | Adjusted model 2 | Adjusted model 3 | Adjusted model 4 |
|---|---|---|---|---|
| **HSV1 (binary)** | 1.14 (0.78, 1.69) p=0.5 | 1.03 (0.69, 1.55) p=0.9 | 1.04 (0.69, 1.58) p=0.9 | 0.98 (0.64, 1.52) p=0.9 |
| **Male (binary)** | 2.22 (1.72, 2.85) p<0.001 | 2.18 (1.67, 2.85) p<0.001 | 1.92 (1.44, 2.56) p<0.001 | 1.28 (0.94, 1.74) p=0.1 |
| **Age (years)** | 1.04 (1.03, 1.05) p<0.001 | 1.04 (1.03, 1.05) p<0.001 | 1.04 (1.03, 1.05) p<0.001 | 1.03 (1.02, 1.04) p<0.001 |
| **BMI (kg/m²)** | | 1.12 (1.09, 1.16) p<0.001 | 1.06 (1.02, 1.10) p=0.005 | 1.06 (1.02, 1.11) p=0.003 |
| **Years of education (years)** | | 0.96 (0.91, 1.01) p=0.1 | 0.96 (0.91, 1.01) p=0.1 | 0.95 (0.90, 1.01) p=0.09 |
| **Ever smoker (binary)** | | 1.12 (0.86, 1.46) p=0.4 | 1.10 (0.84, 1.45) p=0.5 | 1.05 (0.79, 1.40) p=0.7 |
| **Physically active (binary)** | | 0.92 (0.71, 1.21) p=0.6 | 0.96 (0.73, 1.26) p=0.7 | 0.94 (0.71, 1.26) p=0.7 |
| **Parental diabetes (binary)** | | | 1.46 (1.06, 2.00) p=0.02 | 1.33 (0.95, 1.85) p=0.09 |
| **Hypertension (binary)** | | | 1.22 (0.81, 1.83) p=0.3 | 1.16 (0.75, 1.78) p=0.5 |
| **Triglycerides (mmol/l)** | | | 0.88 (0.69, 1.11) p=0.3 | 0.86 (0.67, 1.11) p=0.2 |
| **Total cholesterol / HDL–C (ratio)** | | | 1.28 (1.09, 1.50) p=0.003 | 1.31 (1.11, 1.55) p=0.001 |
| **HOMA–IR (continuous)** | | | 1.42 (1.21, 1.68) p<0.001 | 1.06 (0.90, 1.26) p=0.5 |
| **Fasting glucose (mmol/l)** | | | | 18.53 (10.51, 33.33) p<0.001 |

*ESM Table 6: Individual model coefficients for incidence models for HSV2*

| | Adjusted model 1 | Adjusted model 2 | Adjusted model 3 | Adjusted model 4 |
|---|---|---|---|---|
| **HSV2 (binary)** | 1.57 (1.05, 2.34) p=0.03 | 1.59 (1.05, 2.40) p=0.03 | 1.57 (1.02, 2.40) p=0.04 | 1.59 (1.01, 2.48) p=0.04 |
| **Male (binary)** | 2.22 (1.72, 2.85) p<0.001 | 2.20 (1.68, 2.87) p<0.001 | 1.94 (1.45, 2.59) p<0.001 | 1.29 (0.95, 1.76) p=0.1 |
| **Age (years)** | 1.04 (1.03, 1.05) p<0.001 | 1.04 (1.03, 1.05) p<0.001 | 1.04 (1.03, 1.05) p<0.001 | 1.03 (1.02, 1.04) p<0.001 |
| **BMI (kg/m²)** | | 1.12 (1.09, 1.16) p<0.001 | 1.06 (1.02, 1.10) p=0.005 | 1.06 (1.02, 1.11) p=0.003 |
| **Years of education (years)** | | 0.96 (0.91, 1.01) p=0.1 | 0.96 (0.91, 1.01) p=0.1 | 0.95 (0.90, 1.01) p=0.08 |
| **Ever smoker (binary)** | | 1.10 (0.85, 1.44) p=0.5 | 1.09 (0.83, 1.43) p=0.5 | 1.04 (0.78, 1.39) p=0.8 |
| **Physically active (binary)** | | 0.93 (0.71, 1.21) p=0.6 | 0.96 (0.73, 1.26) p=0.8 | 0.94 (0.71, 1.26) p=0.7 |
| **Parental diabetes (binary)** | | | 1.45 (1.06, 1.99) p=0.02 | 1.33 (0.95, 1.85) p=0.09 |
| **Hypertension (binary)** | | | 1.18 (0.77, 1.78) p=0.4 | 1.11 (0.72, 1.71) p=0.6 |
| **Triglycerides (mmol/l)** | | | 0.87 (0.68, 1.10) p=0.3 | 0.86 (0.67, 1.10) p=0.2 |
| **Total cholesterol / HDL–C (ratio)** | | | 1.28 (1.09, 1.51) p=0.003 | 1.31 (1.11, 1.55) p=0.001 |
| **HOMA–IR (continuous)** | | | 1.42 (1.21, 1.68) p<0.001 | 1.06 (0.90, 1.26) p=0.5 |
| **Fasting glucose (mmol/l)** | | | | 18.58 (10.52, 33.45) p<0.001 |

*ESM Table 7: Individual model coefficients for incidence models for VZV*

| | Adjusted model 1 | Adjusted model 2 | Adjusted model 3 | Adjusted model 4 |
|---|---|---|---|---|
| **VZV (binary)** | 0.87 (0.63, 1.19) p=0.4 | 0.76 (0.55, 1.06) p=0.1 | 0.75 (0.54, 1.05) p=0.09 | 0.74 (0.52, 1.06) p=0.1 |
| **Male (binary)** | 2.17 (1.66, 2.84) p<0.001 | 2.13 (1.60, 2.83) p<0.001 | 1.95 (1.44, 2.66) p<0.001 | 1.27 (0.91, 1.77) p=0.2 |
| **Age (years)** | 1.04 (1.03, 1.05) p<0.001 | 1.04 (1.02, 1.05) p<0.001 | 1.04 (1.03, 1.05) p<0.001 | 1.03 (1.01, 1.04) p<0.001 |
| **BMI (kg/m²)** | | 1.12 (1.08, 1.16) p<0.001 | 1.06 (1.02, 1.10) p=0.007 | 1.07 (1.02, 1.11) p=0.004 |
| **Years of education (years)** | | 0.96 (0.91, 1.02) p=0.2 | 0.97 (0.91, 1.02) p=0.3 | 0.96 (0.90, 1.02) p=0.2 |
| **Ever smoker (binary)** | | 1.10 (0.83, 1.46) p=0.5 | 1.08 (0.81, 1.44) p=0.6 | 1.02 (0.75, 1.38) p=0.9 |
| **Physically active (binary)** | | 0.89 (0.67, 1.18) p=0.4 | 0.91 (0.68, 1.22) p=0.5 | 0.90 (0.66, 1.23) p=0.5 |
| **Parental diabetes (binary)** | | | 1.51 (1.07, 2.10) p=0.02 | 1.38 (0.96, 1.96) p=0.08 |
| **Hypertension (binary)** | | | 1.18 (0.76, 1.83) p=0.5 | 1.12 (0.70, 1.76) p=0.6 |
| **Triglycerides (mmol/l)** | | | 0.89 (0.68, 1.14) p=0.3 | 0.87 (0.67, 1.13) p=0.3 |
| **Total cholesterol / HDL–C (ratio)** | | | 1.23 (1.04, 1.46) p=0.01 | 1.27 (1.06, 1.52) p=0.009 |
| **HOMA–IR (continuous)** | | | 1.41 (1.19, 1.69) p<0.001 | 1.03 (0.86, 1.23) p=0.8 |
| **Fasting glucose (mmol/l)** | | | | 24.89 (13.39, 47.39) p<0.001 |

*ESM Table 8: Individual model coefficients for incidence models for EBV*

| | Adjusted model 1 | Adjusted model 2 | Adjusted model 3 | Adjusted model 4 |
|---|---|---|---|---|
| **EBV (binary)** | 0.55 (0.25, 1.24) p=0.1 | 0.61 (0.26, 1.43) p=0.2 | 0.63 (0.26, 1.51) p=0.3 | 0.59 (0.24, 1.48) p=0.3 |
| **Male (binary)** | 2.18 (1.70, 2.81) p<0.001 | 2.16 (1.65, 2.82) p<0.001 | 1.90 (1.42, 2.53) p<0.001 | 1.27 (0.93, 1.72) p=0.1 |
| **Age (years)** | 1.04 (1.03, 1.06) p<0.001 | 1.04 (1.03, 1.05) p<0.001 | 1.04 (1.03, 1.05) p<0.001 | 1.03 (1.02, 1.04) p<0.001 |
| **BMI (kg/m²)** | | 1.12 (1.09, 1.16) p<0.001 | 1.06 (1.02, 1.10) p=0.006 | 1.06 (1.02, 1.11) p=0.004 |
| **Years of education (years)** | | 0.96 (0.91, 1.01) p=0.1 | 0.96 (0.91, 1.01) p=0.1 | 0.95 (0.90, 1.01) p=0.09 |
| **Ever smoker (binary)** | | 1.13 (0.86, 1.47) p=0.4 | 1.11 (0.85, 1.46) p=0.5 | 1.06 (0.80, 1.41) p=0.7 |
| **Physically active (binary)** | | 0.93 (0.71, 1.21) p=0.6 | 0.96 (0.73, 1.26) p=0.8 | 0.94 (0.71, 1.26) p=0.7 |
| **Parental diabetes (binary)** | | | 1.44 (1.05, 1.98) p=0.02 | 1.32 (0.95, 1.84) p=0.1 |
| **Hypertension (binary)** | | | 1.22 (0.81, 1.84) p=0.3 | 1.16 (0.75, 1.78) p=0.5 |
| **Triglycerides (mmol/l)** | | | 0.88 (0.69, 1.12) p=0.3 | 0.87 (0.68, 1.12) p=0.3 |
| **Total cholesterol / HDL–C (ratio)** | | | 1.28 (1.09, 1.50) p=0.003 | 1.31 (1.11, 1.55) p=0.002 |
| **HOMA–IR (continuous)** | | | 1.42 (1.21, 1.67) p<0.001 | 1.06 (0.90, 1.26) p=0.5 |
| **Fasting glucose (mmol/l)** | | | | 18.58 (10.53, 33.42) p<0.001 |

*ESM Table 9: Individual model coefficients for incidence models for CMV*

| | Adjusted model 1 | Adjusted model 2 | Adjusted model 3 | Adjusted model 4 |
|---|---|---|---|---|
| **CMV (binary)** | 1.37 (1.06, 1.77) p=0.02 | 1.35 (1.03, 1.76) p=0.03 | 1.37 (1.04, 1.79) p=0.02 | 1.33 (1.00, 1.78) p=0.05 |
| **Male (binary)** | 2.28 (1.77, 2.95) p<0.001 | 2.26 (1.73, 2.97) p<0.001 | 2.00 (1.50, 2.68) p<0.001 | 1.33 (0.98, 1.82) p=0.07 |
| **Age (years)** | 1.04 (1.03, 1.05) p<0.001 | 1.04 (1.02, 1.05) p<0.001 | 1.04 (1.03, 1.05) p<0.001 | 1.03 (1.01, 1.04) p<0.001 |
| **BMI (kg/m²)** | | 1.12 (1.09, 1.16) p<0.001 | 1.06 (1.02, 1.10) p=0.006 | 1.06 (1.02, 1.11) p=0.004 |
| **Years of education (years)** | | 0.96 (0.91, 1.01) p=0.09 | 0.96 (0.91, 1.01) p=0.1 | 0.95 (0.90, 1.01) p=0.08 |
| **Ever smoker (binary)** | | 1.10 (0.84, 1.44) p=0.5 | 1.09 (0.83, 1.43) p=0.6 | 1.03 (0.78, 1.38) p=0.8 |
| **Physically active (binary)** | | 0.93 (0.71, 1.22) p=0.6 | 0.97 (0.74, 1.28) p=0.8 | 0.96 (0.72, 1.28) p=0.8 |
| **Parental diabetes (binary)** | | | 1.44 (1.05, 1.98) p=0.02 | 1.32 (0.95, 1.84) p=0.1 |
| **Hypertension (binary)** | | | 1.26 (0.83, 1.89) p=0.3 | 1.20 (0.78, 1.84) p=0.4 |
| **Triglycerides (mmol/l)** | | | 0.87 (0.68, 1.11) p=0.3 | 0.86 (0.67, 1.10) p=0.2 |
| **Total cholesterol / HDL–C (ratio)** | | | 1.27 (1.09, 1.50) p=0.003 | 1.31 (1.11, 1.56) p=0.001 |
| **HOMA–IR (continuous)** | | | 1.43 (1.22, 1.69) p<0.001 | 1.07 (0.90, 1.27) p=0.4 |
| **Fasting glucose (mmol/l)** | | | | 18.39 (10.41, 33.12) p<0.001 |

*ESM Table 10: Individual model coefficients for incidence models for HHV6*

| | Adjusted model 1 | Adjusted model 2 | Adjusted model 3 | Adjusted model 4 |
|---|---|---|---|---|
| **HHV6 (binary)** | 1.20 (0.93, 1.55) p=0.2 | 1.21 (0.93, 1.57) p=0.2 | 1.18 (0.90, 1.54) p=0.2 | 1.26 (0.95, 1.67) p=0.1 |
| **Male (binary)** | 2.22 (1.73, 2.86) p<0.001 | 2.19 (1.68, 2.87) p<0.001 | 1.93 (1.45, 2.58) p<0.001 | 1.30 (0.95, 1.76) p=0.1 |
| **Age (years)** | 1.04 (1.03, 1.06) p<0.001 | 1.04 (1.03, 1.05) p<0.001 | 1.04 (1.03, 1.05) p<0.001 | 1.03 (1.02, 1.04) p<0.001 |
| **BMI (kg/m²)** | | 1.12 (1.09, 1.16) p<0.001 | 1.06 (1.02, 1.10) p=0.005 | 1.06 (1.02, 1.11) p=0.003 |
| **Years of education (years)** | | 0.96 (0.91, 1.01) p=0.1 | 0.96 (0.91, 1.01) p=0.1 | 0.95 (0.90, 1.01) p=0.08 |
| **Ever smoker (binary)** | | 1.11 (0.85, 1.45) p=0.4 | 1.10 (0.84, 1.44) p=0.5 | 1.04 (0.78, 1.39) p=0.8 |
| **Physically active (binary)** | | 0.93 (0.71, 1.21) p=0.6 | 0.96 (0.73, 1.27) p=0.8 | 0.95 (0.71, 1.27) p=0.7 |
| **Parental diabetes (binary)** | | | 1.46 (1.06, 2.00) p=0.02 | 1.34 (0.96, 1.86) p=0.09 |
| **Hypertension (binary)** | | | 1.22 (0.81, 1.84) p=0.3 | 1.16 (0.75, 1.78) p=0.5 |
| **Triglycerides (mmol/l)** | | | 0.88 (0.69, 1.12) p=0.3 | 0.87 (0.68, 1.11) p=0.3 |
| **Total cholesterol / HDL–C (ratio)** | | | 1.27 (1.08, 1.50) p=0.003 | 1.31 (1.11, 1.55) p=0.002 |
| **HOMA–IR (continuous)** | | | 1.42 (1.21, 1.68) p<0.001 | 1.06 (0.89, 1.25) p=0.5 |
| **Fasting glucose (mmol/l)** | | | | 18.97 (10.73, 34.22) p<0.001 |

*ESM Table 11: Individual model coefficients for incidence models for HHV7*

| | Adjusted model 1 | Adjusted model 2 | Adjusted model 3 | Adjusted model 4 |
|---|---|---|---|---|
| **HHV7 (binary)** | 0.93 (0.66, 1.33) p=0.7 | 0.95 (0.66, 1.37) p=0.8 | 0.91 (0.63, 1.32) p=0.6 | 0.99 (0.68, 1.46) p=1 |
| **Male (binary)** | 2.20 (1.71, 2.83) p<0.001 | 2.17 (1.66, 2.84) p<0.001 | 1.91 (1.43, 2.55) p<0.001 | 1.28 (0.94, 1.74) p=0.1 |
| **Age (years)** | 1.04 (1.03, 1.05) p<0.001 | 1.04 (1.03, 1.05) p<0.001 | 1.04 (1.03, 1.05) p<0.001 | 1.03 (1.02, 1.04) p<0.001 |
| **BMI (kg/m²)** | | 1.12 (1.09, 1.16) p<0.001 | 1.06 (1.02, 1.10) p=0.005 | 1.06 (1.02, 1.11) p=0.003 |
| **Years of education (years)** | | 0.96 (0.91, 1.01) p=0.1 | 0.96 (0.91, 1.01) p=0.1 | 0.95 (0.90, 1.01) p=0.09 |
| **Ever smoker (binary)** | | 1.12 (0.86, 1.46) p=0.4 | 1.10 (0.84, 1.45) p=0.5 | 1.05 (0.79, 1.40) p=0.7 |
| **Physically active (binary)** | | 0.92 (0.71, 1.21) p=0.6 | 0.96 (0.73, 1.26) p=0.8 | 0.94 (0.71, 1.26) p=0.7 |
| **Parental diabetes (binary)** | | | 1.46 (1.06, 2.00) p=0.02 | 1.33 (0.96, 1.85) p=0.09 |
| **Hypertension (binary)** | | | 1.23 (0.81, 1.85) p=0.3 | 1.16 (0.75, 1.78) p=0.5 |
| **Triglycerides (mmol/l)** | | | 0.88 (0.69, 1.12) p=0.3 | 0.86 (0.67, 1.11) p=0.2 |
| **Total cholesterol / HDL–C (ratio)** | | | 1.28 (1.09, 1.50) p=0.003 | 1.31 (1.11, 1.55) p=0.001 |
| **HOMA–IR (continuous)** | | | 1.42 (1.21, 1.68) p<0.001 | 1.06 (0.90, 1.26) p=0.5 |
| **Fasting glucose (mmol/l)** | | | | 18.51 (10.49, 33.31) p<0.001 |

## ESM Figures

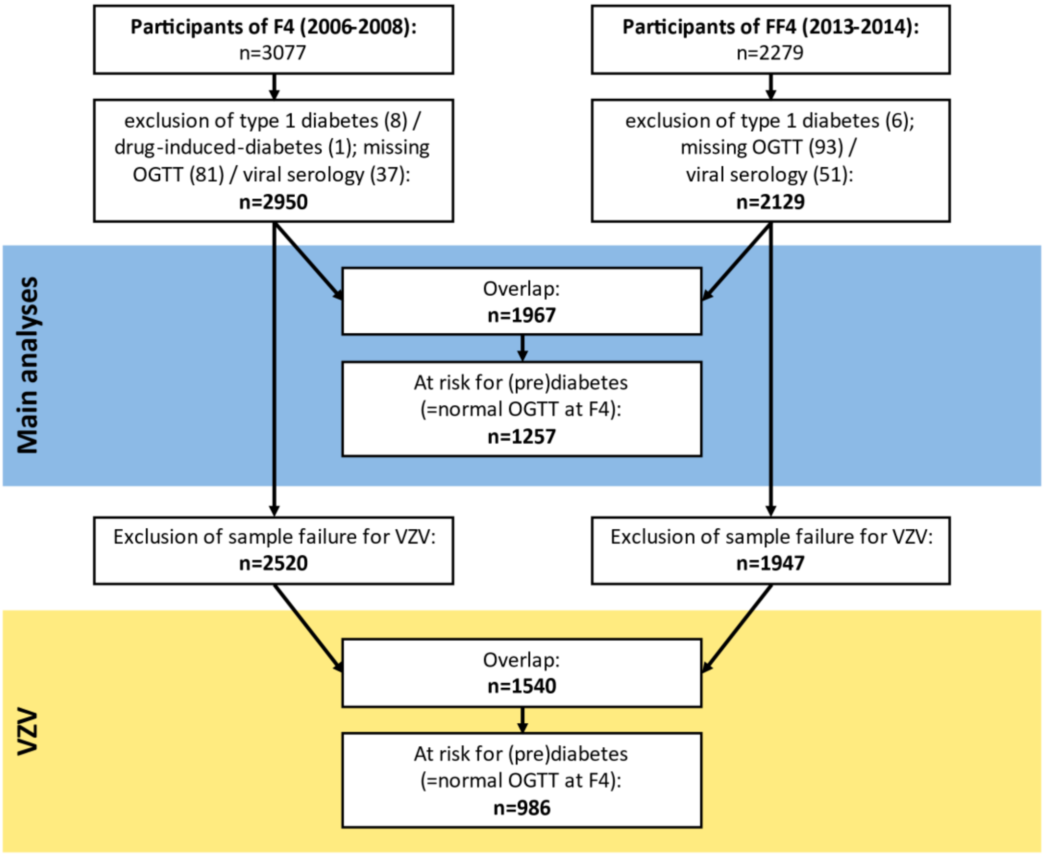


*ESM Figure 1: Flowchart of participant selection*

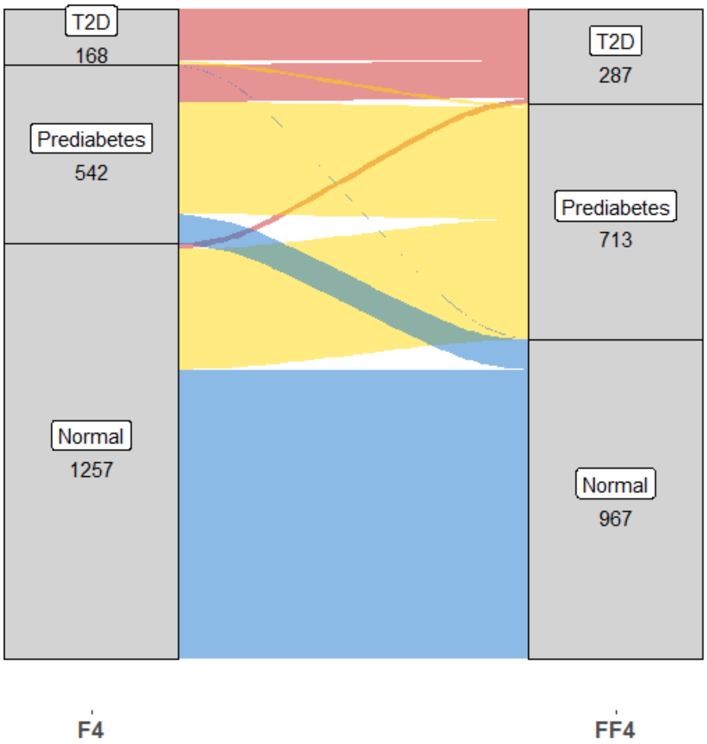


*ESM Figure 2: Comparing glucose tolerance status at baseline (F4) and follow–up (FF4) with on average 6.5 years in between (n=1967)*

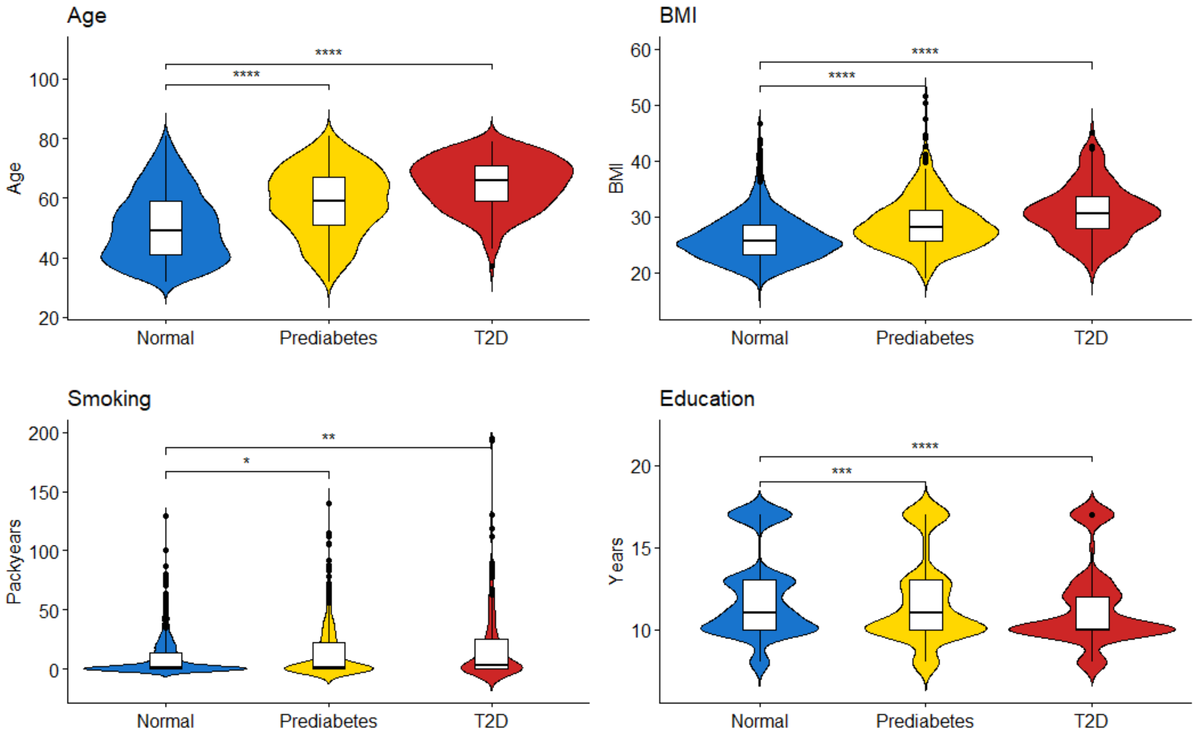


*ESM Figure 3: T2D risk factors age, overweight, smoking, and poor–education at F4 timepoint*

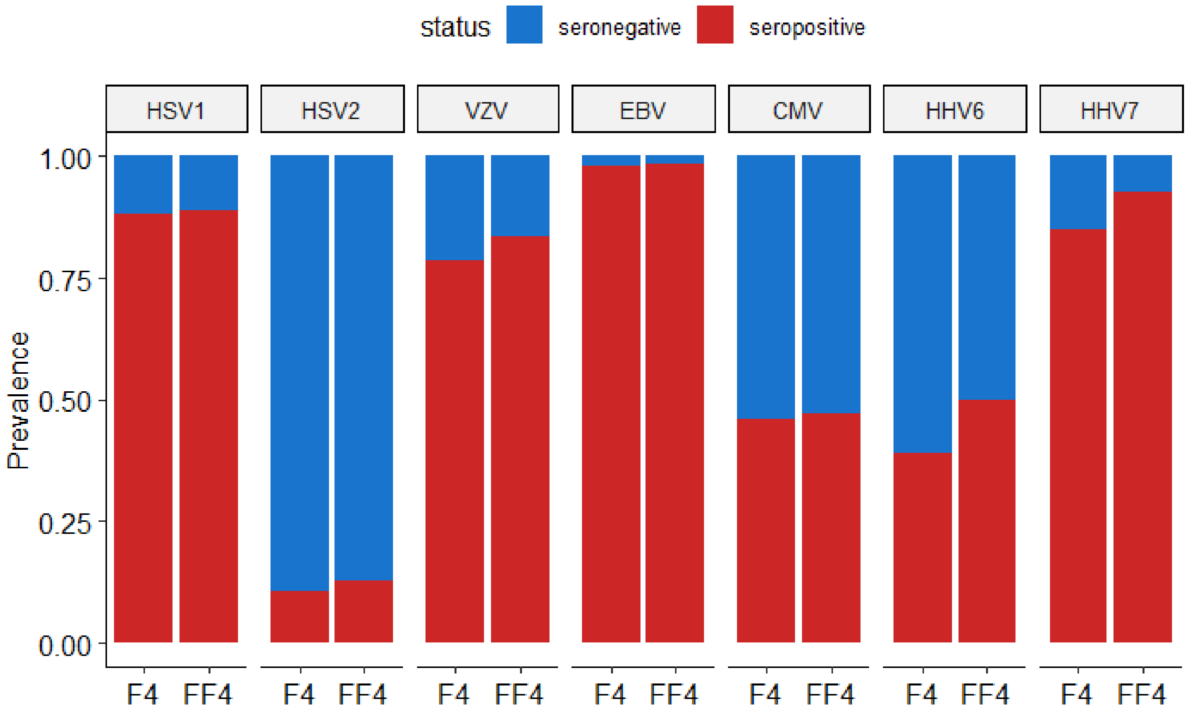


*ESM Figure 4: Viral seroprevalences at baseline (F4) and follow–up (FF4) for the seven herpesviruses under investigation (n=1967, except for VZV n=1540)*

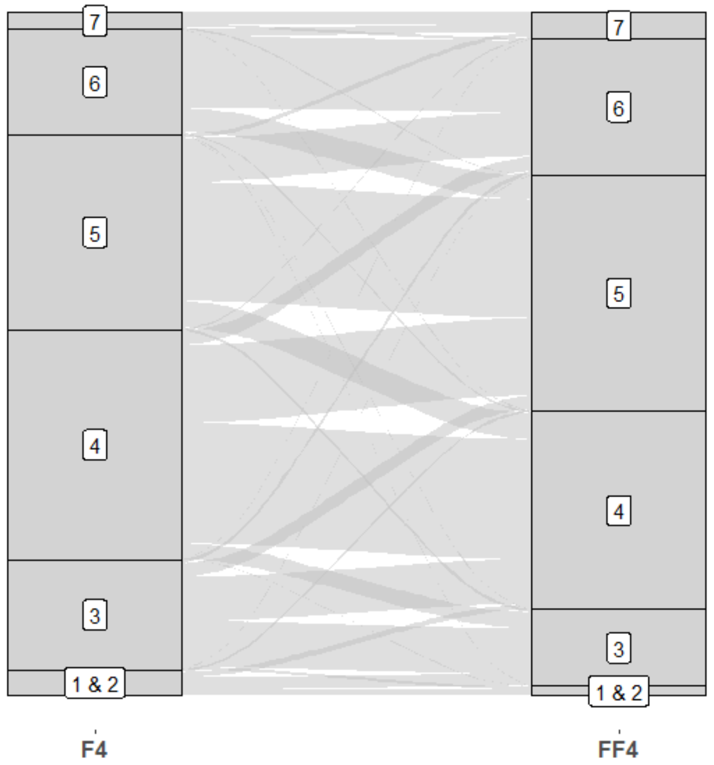


*ESM Figure 5: Changes of number of seropositive viruses per person from F4 to FF4 (n=1540) (B).*

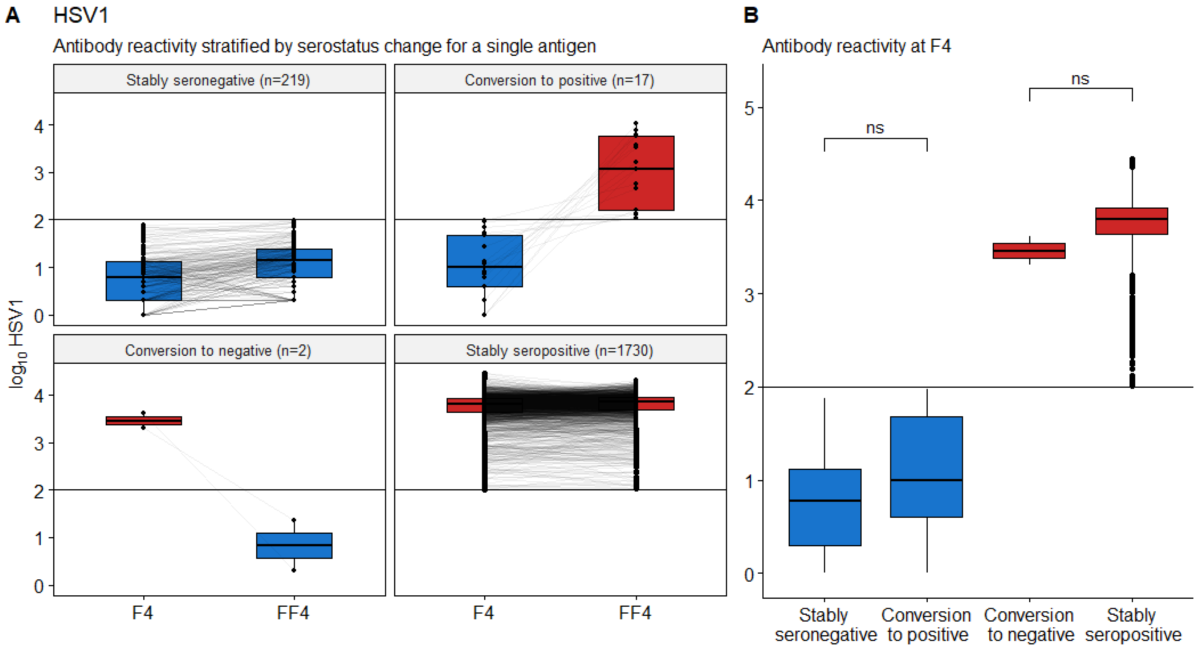


*ESM Figure 6: Overview of serostatus change for HSV1 gG antigen*

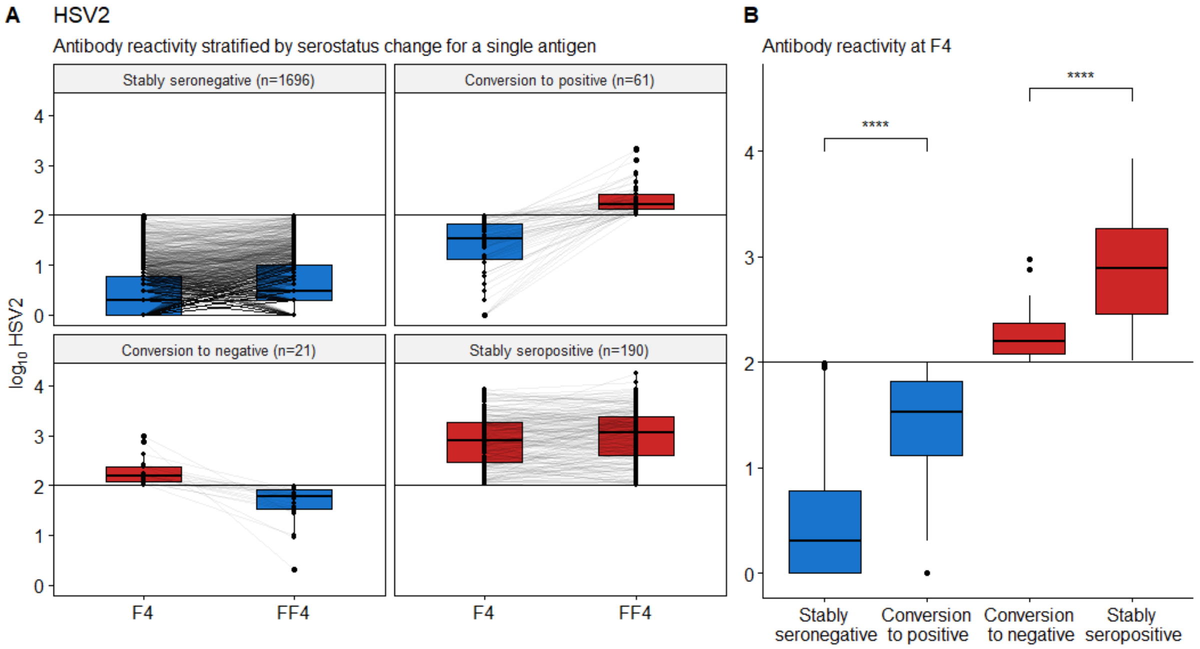


*ESM Figure 7: Overview of serostatus change for HSV2 mgG unique antigen*

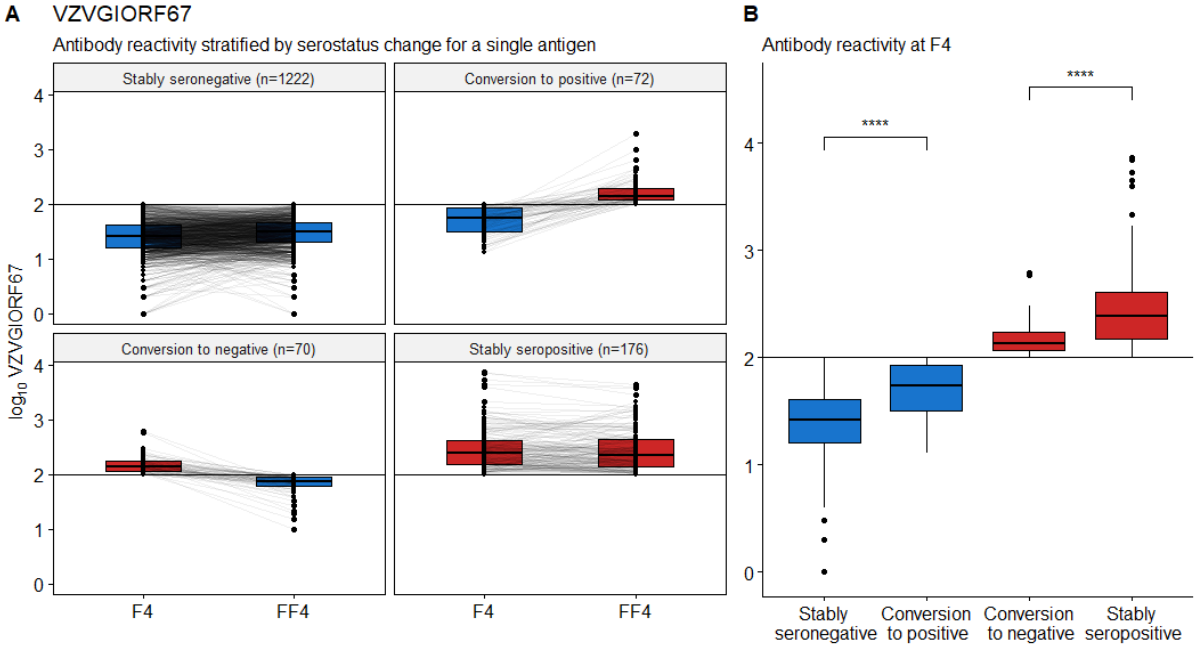


*ESM Figure 8: Overview of serostatus change for VZV gI(ORF67) antigen*

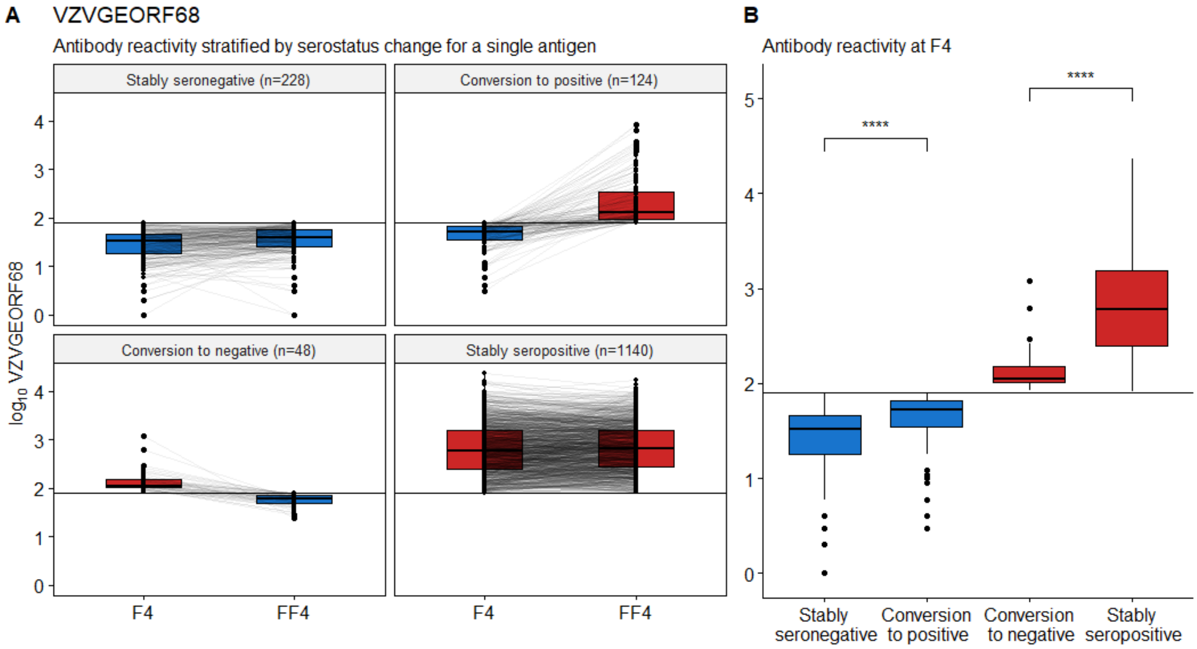


*ESM Figure 9: Overview of serostatus change for VZV gE(ORF68) antigen*

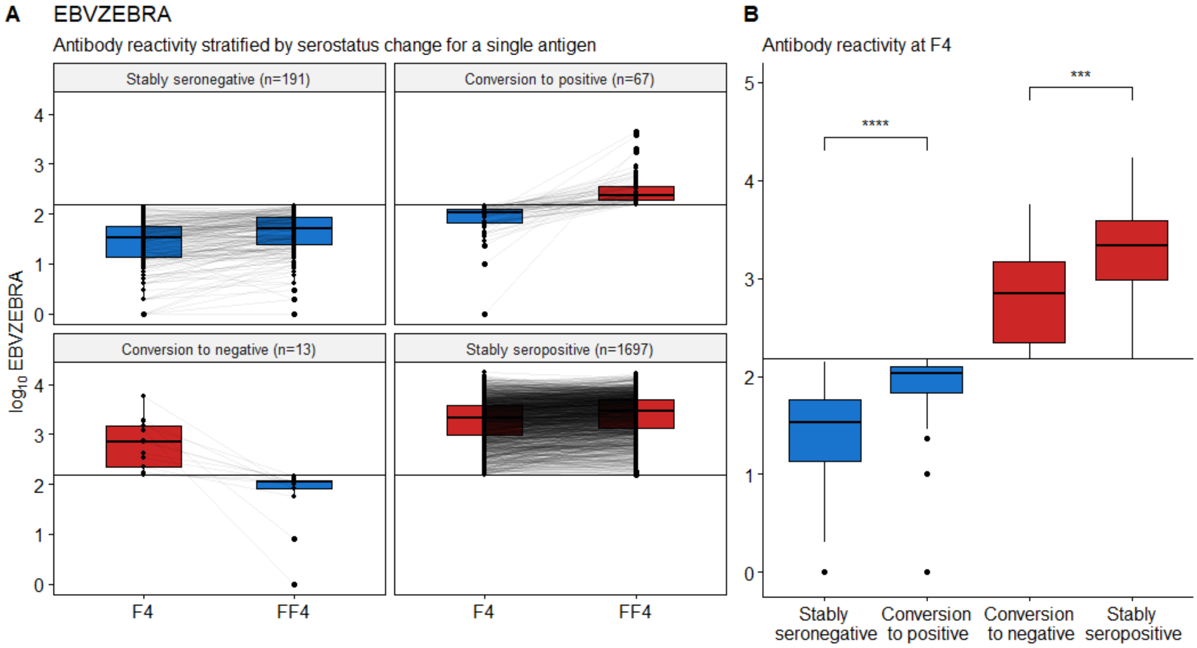


*ESM Figure 10: Overview of serostatus change for EBV Zebra antigen*

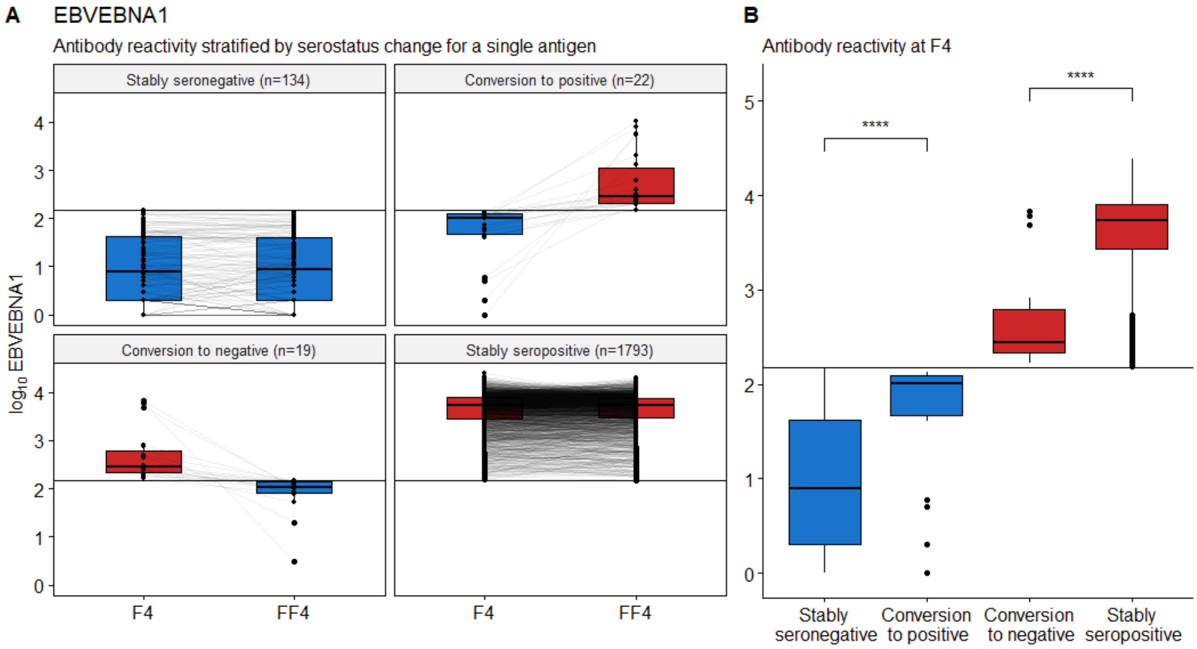


*ESM Figure 11: Overview of serostatus change for EBV EBNA–1 antigen*

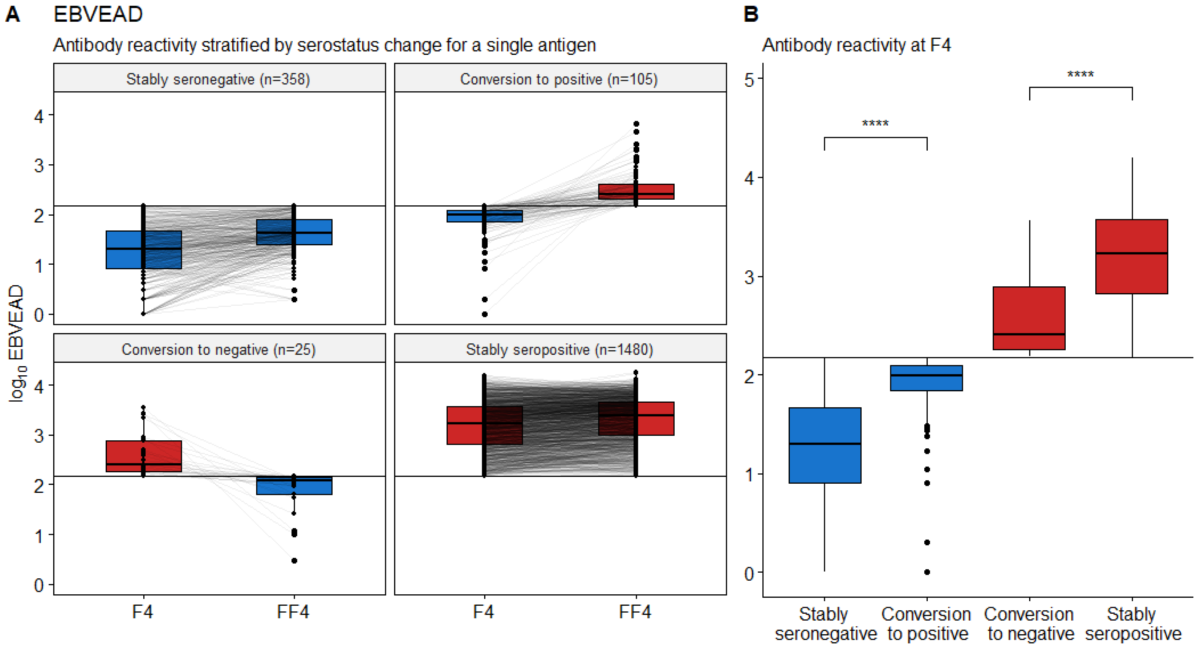


*ESM Figure 12: Overview of serostatus change for EBV EA–D antigen*

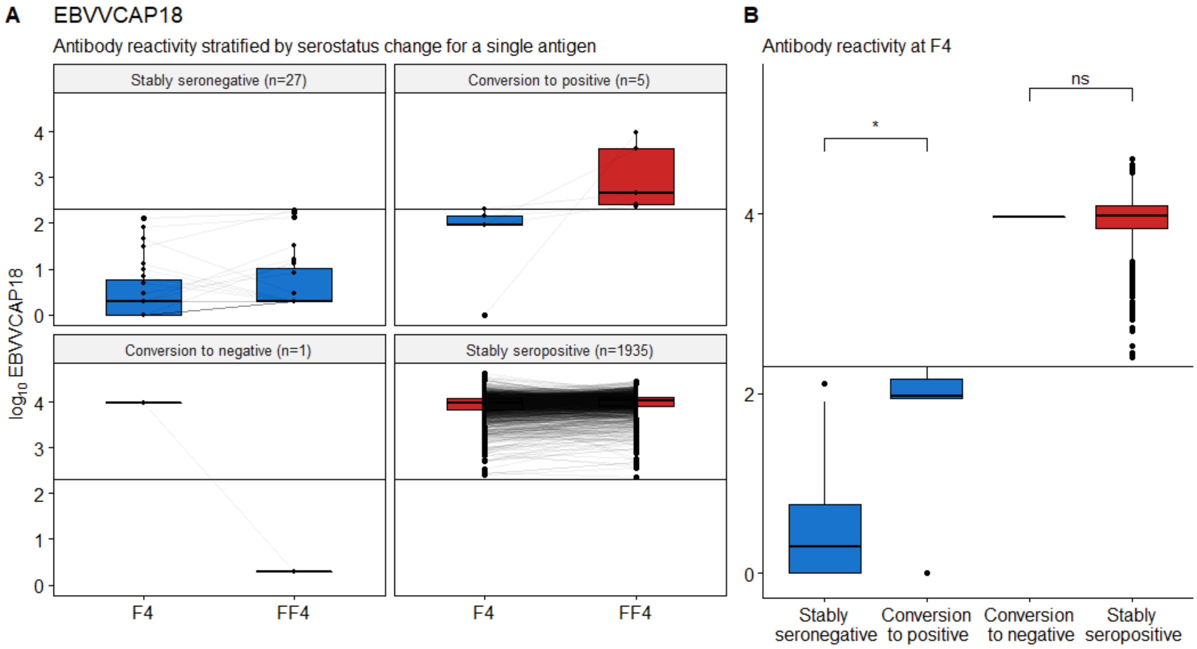


*ESM Figure 13: Overview of serostatus change for EBV VCA p18 antigen*

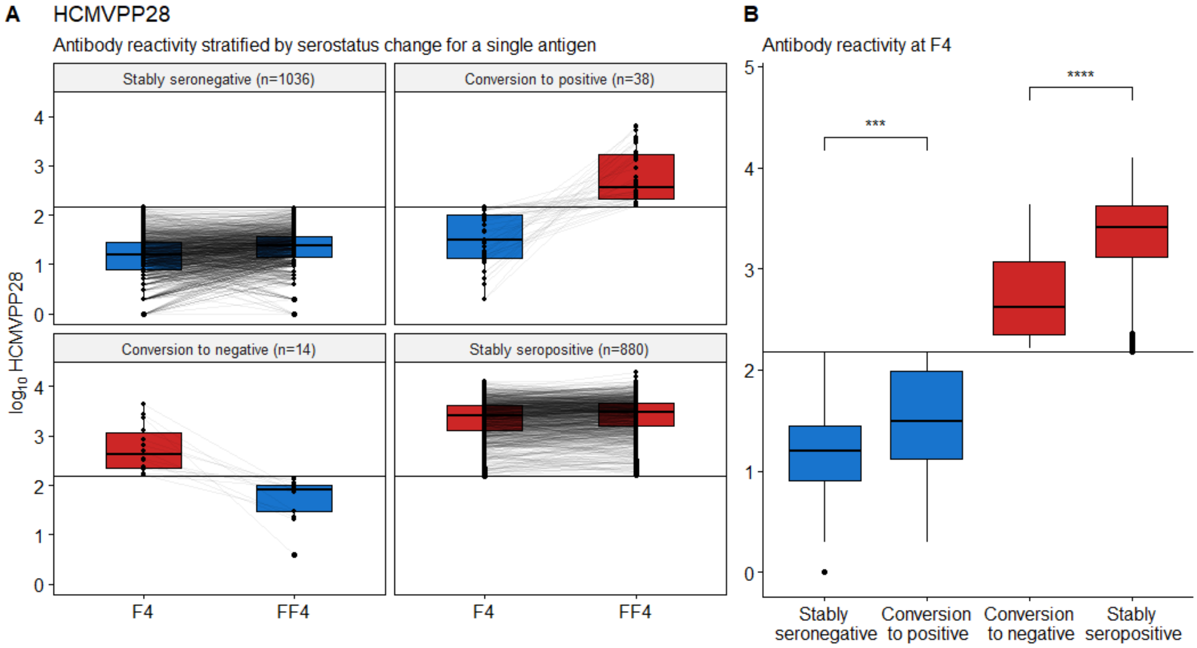


*ESM Figure 14: Overview of serostatus change for CMV pp28 antigen*

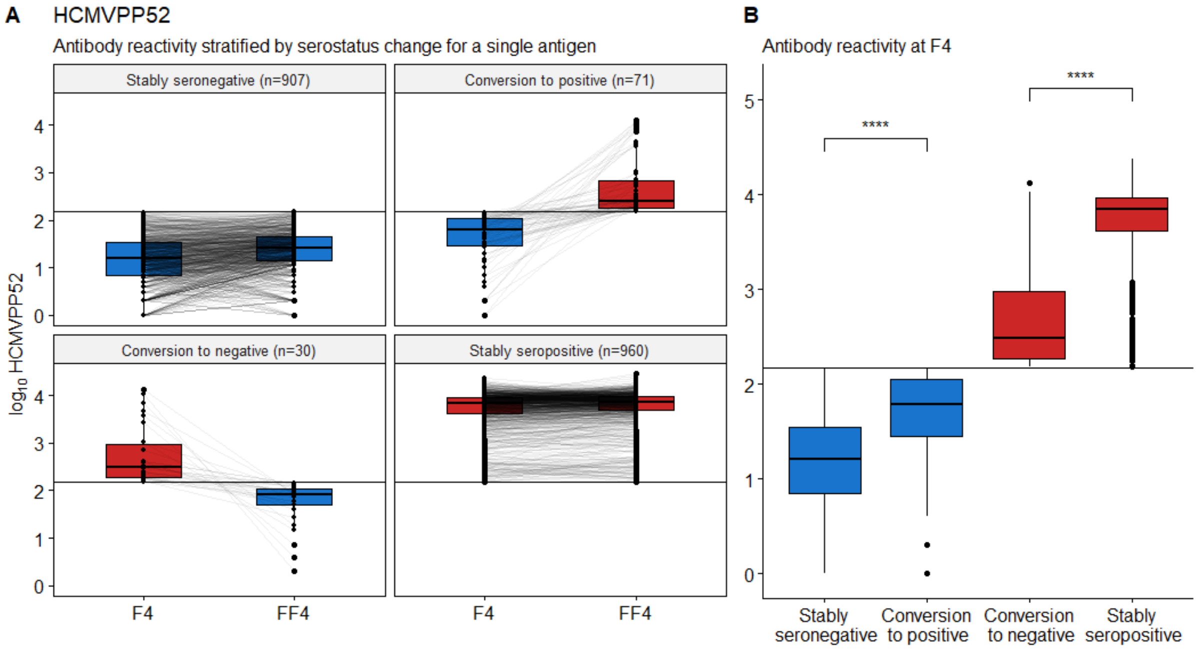


*ESM Figure 15: Overview of serostatus change for CMV pp52 antigen*

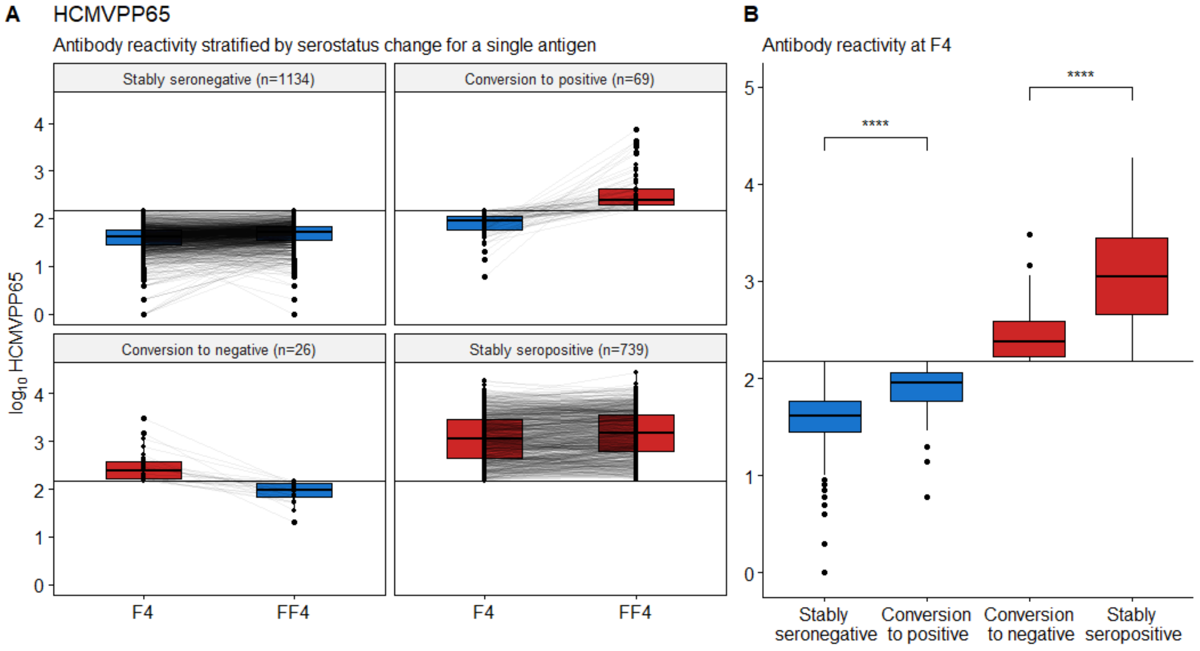


*ESM Figure 16: Overview of serostatus change for CMV pp65 antigen*

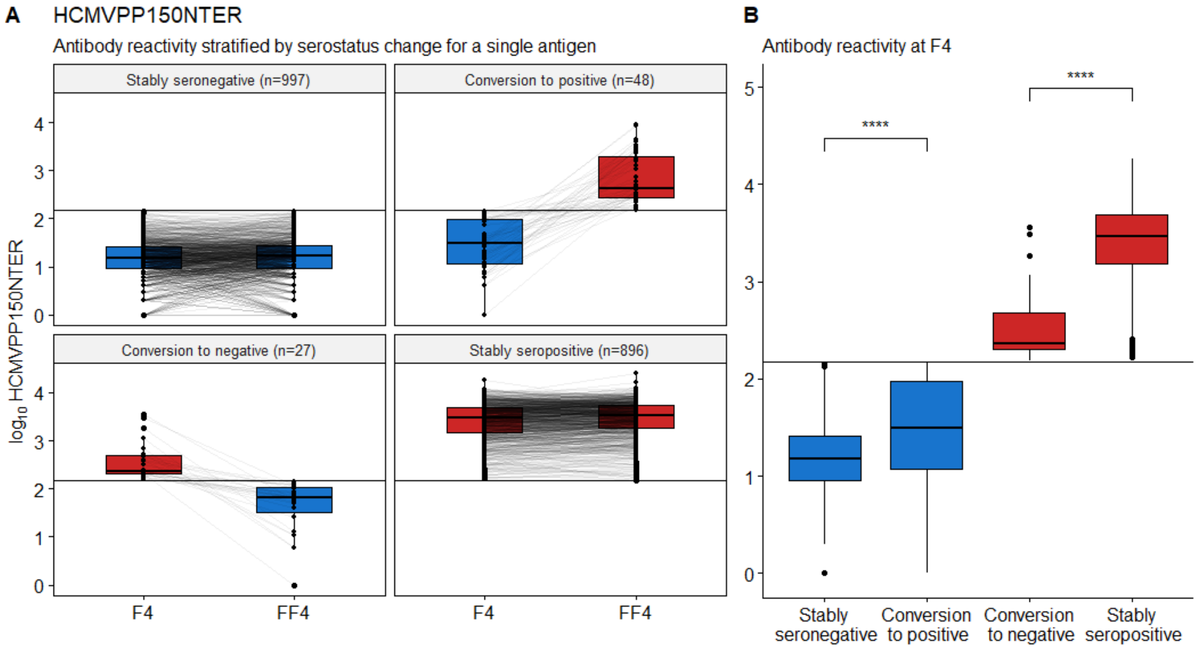


*ESM Figure 17: Overview of serostatus change for CMV pp150 Nter antigen*

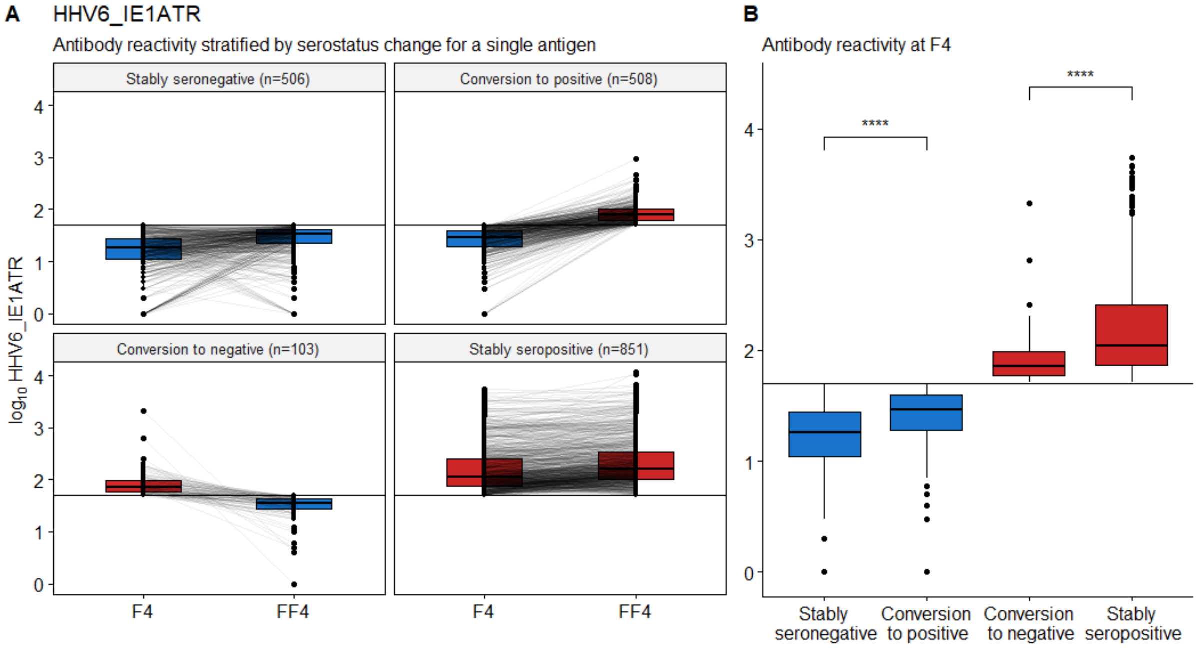


*ESM Figure 18: Overview of serostatus change for HHV6 IE1A trunc. antigen*

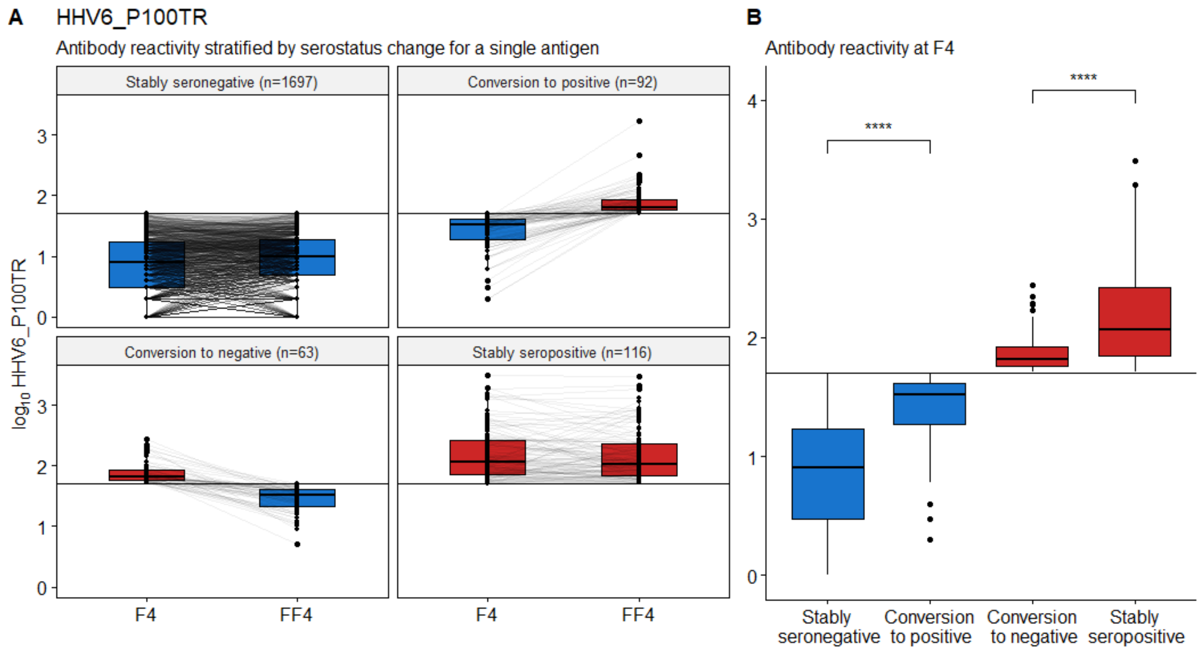


*ESM Figure 19: Overview of serostatus change for HHV6 p100 trunc. antigen*

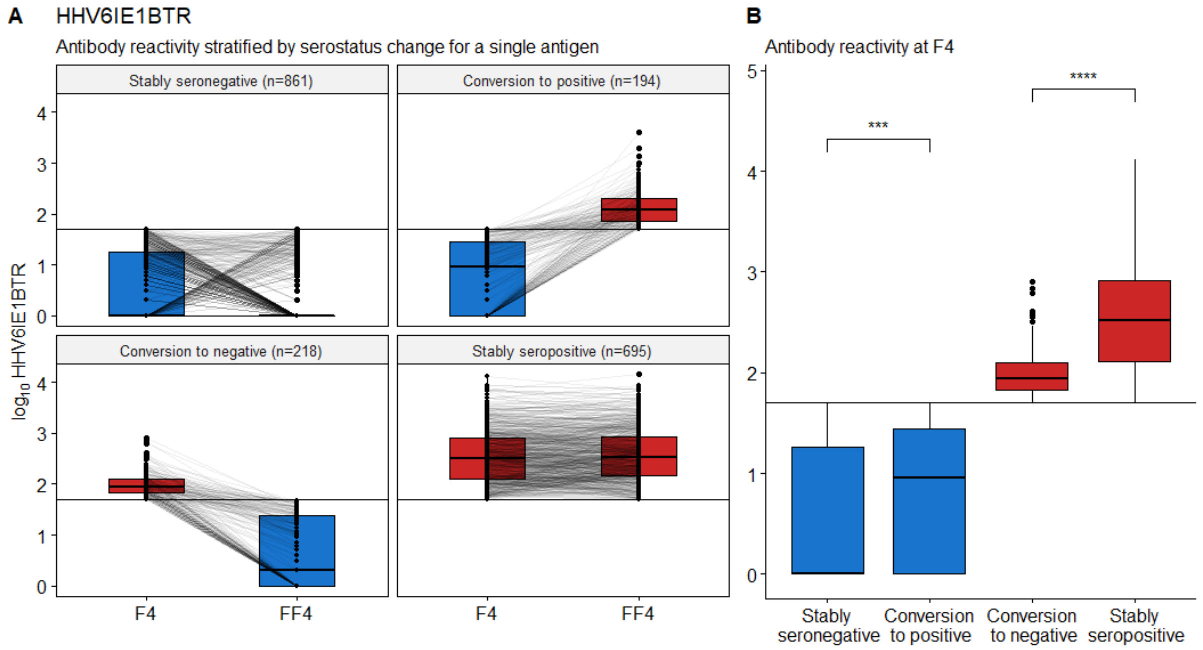


*ESM Figure 20: Overview of serostatus change for HHV6 IE1B trunc. antigen*

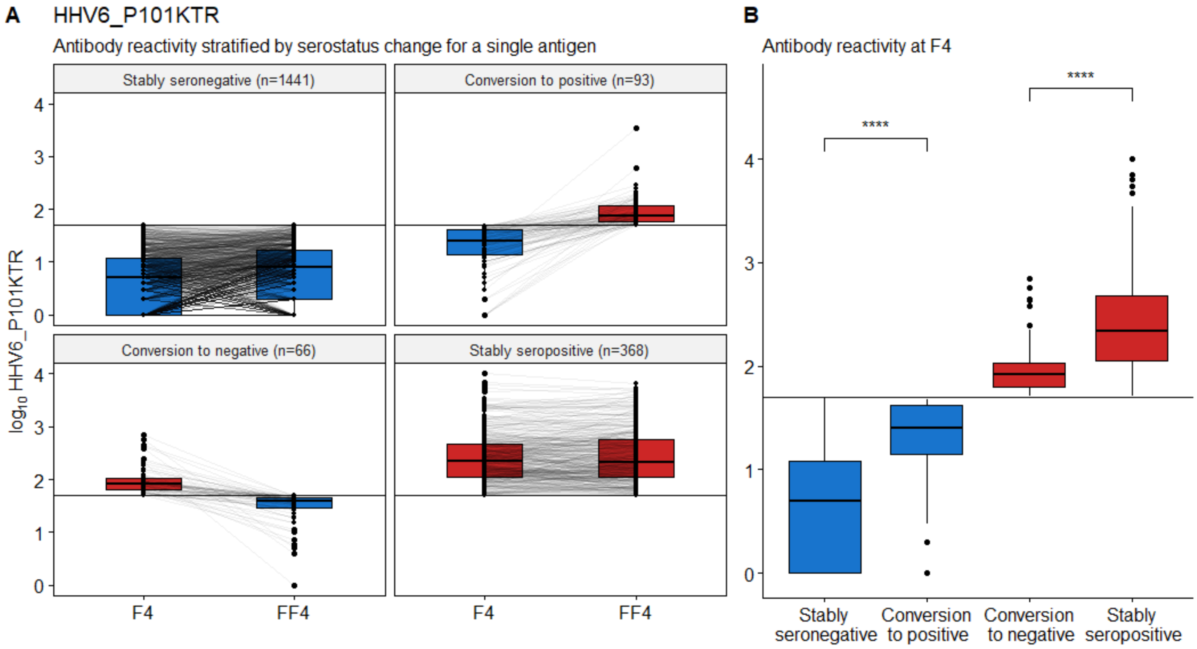


*ESM Figure 21: Overview of serostatus change for HHV6 p101K trunc. antigen*

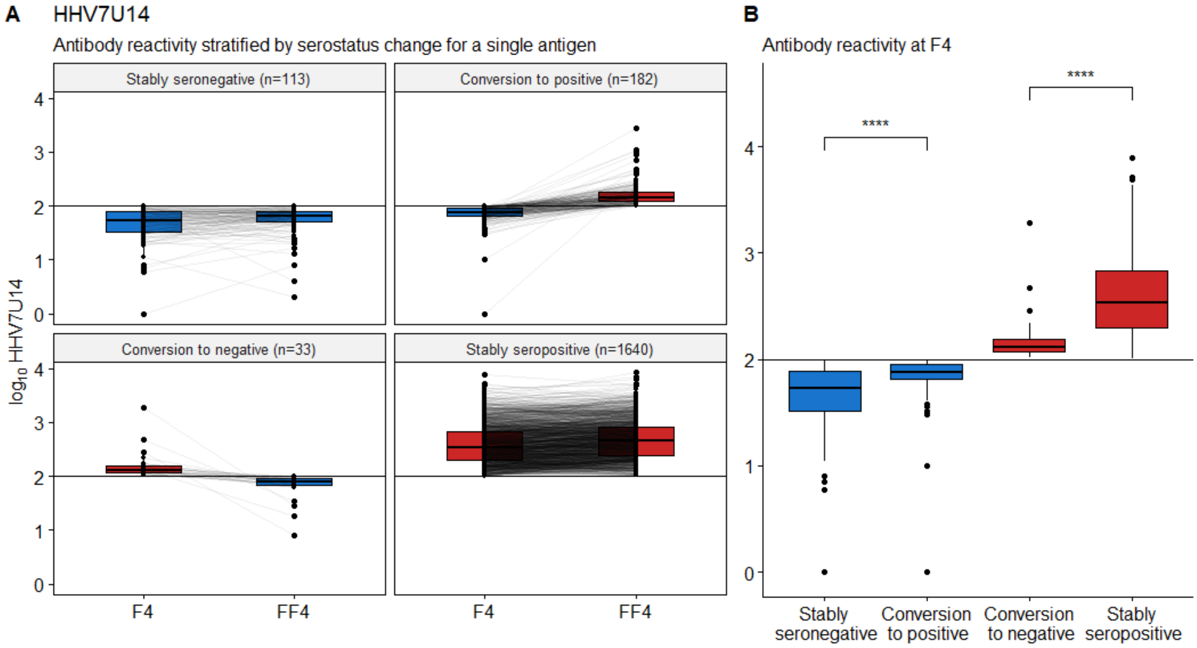


*ESM Figure 22: Overview of serostatus change for HHV7 U14 antigen*

| At risk at F4 n=1257 | 168 HSV1- | 1089 HSV1+ | | |
|---|---|---|---|---|
| Normal glucose tolerance at FF4 | 125 (74.4%) | 751 (69.0%) | | |
| Incident (pre)diabetes at FF4 | 43 (25.6%) | 338 (31.0%) | OR | 1.31 |
| Male | 48.8% | 41.0% | OR.adj | 1.41 |
| Mean age at F4 | 46.0 | 51.2 | OR.adj | 1.06 |
| Mean BMI at F4 | 25.6 | 26.3 | OR.adj | 1.21 |
| Mean years of education | 13.3 | 11.9 | OR.adj | 1.17 |
| Ever smoker at F4 | 56.0% | 58.3% | OR.adj | 1.30 |
| Physically active at F4 | 60.7% | 61.2% | OR.adj | 1.31 |
| Parental diabetes | 22.6% | 20.9% | OR.adj | 1.32 |
| Hypertension (>140/90mmHg) at F4 | 7.1% | 10.9% | OR.adj | 1.28 |
| Mean triglycerides (mmol/l) at F4 | 1.09 | 1.18 | OR.adj | 1.26 |
| Mean cholesterol/HDL at F4 | 3.79 | 3.84 | OR.adj | 1.30 |
| Mean HOMA-IR at F4 | 1.76 | 1.88 | OR.adj | 1.24 |
| Mean fasting glucose (mmol/l) at F4 | 4.9 | 5.0 | OR.adj | 1.15 |
| All confounders combined | | | OR.adj | 0.98 |

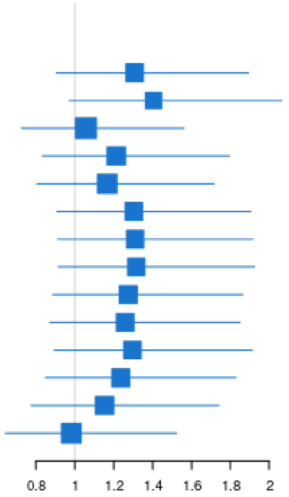


*ESM Figure 23: Contingency table and contribution of individual covariates for the association of HSV1 with (pre)diabetes incidence*

| At risk at F4 n=1257 | 1134 HSV2- | 123 HSV2+ | | |
|---|---|---|---|---|
| Normal glucose tolerance at FF4 | 803 (70.8%) | 73 (59.3%) | | |
| Incident (pre)diabetes at FF4 | 331 (29.2%) | 50 (40.7%) | OR | 1.66 |
| Male | 42.1% | 41.5% | OR.adj | 1.70 |
| Mean age at F4 | 50.3 | 52.8 | OR.adj | 1.53 |
| Mean BMI at F4 | 26.2 | 26.3 | OR.adj | 1.70 |
| Mean years of education | 12.1 | 12.1 | OR.adj | 1.67 |
| Ever smoker at F4 | 57.1% | 65.9% | OR.adj | 1.64 |
| Physically active at F4 | 61.2% | 61.0% | OR.adj | 1.66 |
| Parental diabetes | 21.0% | 22.8% | OR.adj | 1.66 |
| Hypertension (>140/90mmHg) at F4 | 9.6% | 17.9% | OR.adj | 1.58 |
| Mean triglycerides (mmol/l) at F4 | 1.15 | 1.27 | OR.adj | 1.59 |
| Mean cholesterol/HDL at F4 | 3.83 | 3.86 | OR.adj | 1.69 |
| Mean HOMA-IR at F4 | 1.86 | 1.89 | OR.adj | 1.68 |
| Mean fasting glucose (mmol/l) at F4 | 5.0 | 5.0 | OR.adj | 1.66 |
| All confounders combined | | | OR.adj | 1.59 |

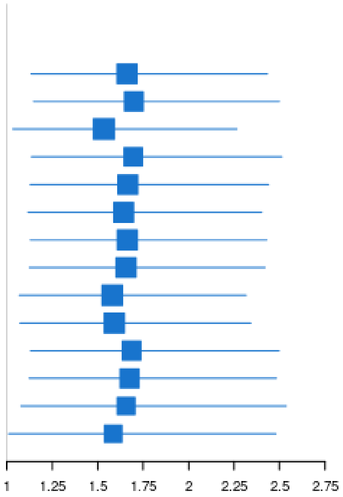


*ESM Figure 24: Contingency table and contribution of individual covariates for the association of HSV2 with (pre)diabetes incidence*

| At risk at F4 n=1091 | 251 VZV- | 840 VZV+ | | |
|---|---|---|---|---|
| Normal glucose tolerance at FF4 | 169 (67.3%) | 579 (68.9%) | | |
| Incident (pre)diabetes at FF4 | 82 (32.7%) | 261 (31.1%) | OR | 0.93 |
| Male | 35.1% | 44.4% | OR.adj | 0.86 |
| Mean age at F4 | 51.2 | 50.8 | OR.adj | 0.94 |
| Mean BMI at F4 | 25.5 | 26.4 | OR.adj | 0.81 |
| Mean years of education | 12.1 | 12.1 | OR.adj | 0.92 |
| Ever smoker at F4 | 53.8% | 58.9% | OR.adj | 0.92 |
| Physically active at F4 | 64.5% | 60.0% | OR.adj | 0.92 |
| Parental diabetes | 20.3% | 21.2% | OR.adj | 0.93 |
| Hypertension (>140/90mmHg) at F4 | 9.2% | 11.0% | OR.adj | 0.92 |
| Mean triglycerides (mmol/l) at F4 | 1.13 | 1.18 | OR.adj | 0.90 |
| Mean cholesterol/HDL at F4 | 3.77 | 3.86 | OR.adj | 0.89 |
| Mean HOMA-IR at F4 | 1.75 | 1.90 | OR.adj | 0.85 |
| Mean fasting glucose (mmol/l) at F4 | 4.9 | 5.0 | OR.adj | 0.82 |
| All confounders combined | | | OR.adj | 0.74 |

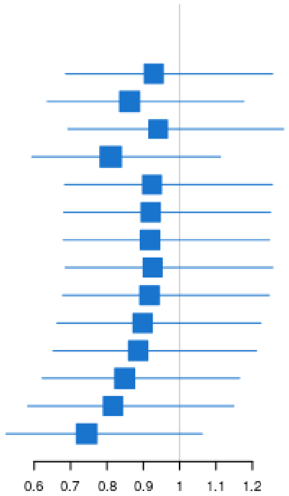


*ESM Figure 25: Contingency table and contribution of individual covariates for the association of VZV with (pre)diabetes incidence*

| At risk at F4 n=1257 | 27 EBV- | 1230 EBV+ | | |
|---|---|---|---|---|
| Normal glucose tolerance at FF4 | 15 (55.6%) | 861 (70.0%) | | |
| Incident (pre)diabetes at FF4 | 12 (44.4%) | 369 (30.0%) | OR | 0.54 |
| Male | 63.0% | 41.5% | OR.adj | 0.62 |
| Mean age at F4 | 48.5 | 50.6 | OR.adj | 0.47 |
| Mean BMI at F4 | 27.5 | 26.2 | OR.adj | 0.61 |
| Mean years of education | 12.7 | 12.1 | OR.adj | 0.50 |
| Ever smoker at F4 | 40.7% | 58.4% | OR.adj | 0.52 |
| Physically active at F4 | 51.9% | 61.4% | OR.adj | 0.55 |
| Parental diabetes | 33.3% | 20.9% | OR.adj | 0.55 |
| Hypertension (>140/90mmHg) at F4 | 11.1% | 10.4% | OR.adj | 0.53 |
| Mean triglycerides (mmol/l) at F4 | 1.04 | 1.17 | OR.adj | 0.49 |
| Mean cholesterol/HDL at F4 | 3.90 | 3.83 | OR.adj | 0.53 |
| Mean HOMA-IR at F4 | 2.08 | 1.85 | OR.adj | 0.59 |
| Mean fasting glucose (mmol/l) at F4 | 5.0 | 5.0 | OR.adj | 0.57 |
| All confounders combined | | | OR.adj | 0.59 |

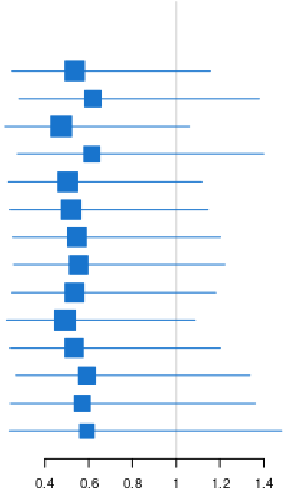


*ESM Figure 26: Contingency table and contribution of individual covariates for the association of EBV with (pre)diabetes incidence*

| At risk at F4 n=1257 | 697 CMV- | 560 CMV+ | | |
|---|---|---|---|---|
| Normal glucose tolerance at FF4 | 511 (73.3%) | 365 (65.2%) | | |
| Incident (pre)diabetes at FF4 | 186 (26.7%) | 195 (34.8%) | OR | 1.47 |
| Male | 46.3% | 36.6% | OR.adj | 1.62 |
| Mean age at F4 | 48.5 | 53.0 | OR.adj | 1.25 |
| Mean BMI at F4 | 26.1 | 26.4 | OR.adj | 1.43 |
| Mean years of education | 12.2 | 12.0 | OR.adj | 1.45 |
| Ever smoker at F4 | 56.0% | 60.5% | OR.adj | 1.46 |
| Physically active at F4 | 62.8% | 59.1% | OR.adj | 1.46 |
| Parental diabetes | 19.9% | 22.7% | OR.adj | 1.46 |
| Hypertension (>140/90mmHg) at F4 | 11.3% | 9.3% | OR.adj | 1.50 |
| Mean triglycerides (mmol/l) at F4 | 1.16 | 1.18 | OR.adj | 1.47 |
| Mean cholesterol/HDL at F4 | 3.82 | 3.84 | OR.adj | 1.49 |
| Mean HOMA-IR at F4 | 1.86 | 1.85 | OR.adj | 1.51 |
| Mean fasting glucose (mmol/l) at F4 | 4.9 | 5.0 | OR.adj | 1.44 |
| All confounders combined | | | OR.adj | 1.33 |

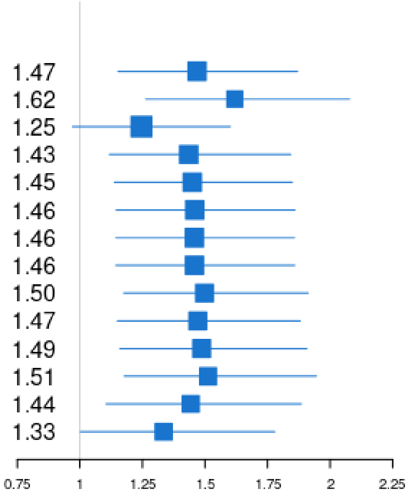


*ESM Figure 27: Contingency table and contribution of individual covariates for the association of CMV with (pre)diabetes incidence*

| At risk at F4 n=1257 | 765 HHV6- | 492 HHV6+ | | |
|---|---|---|---|---|
| Normal glucose tolerance at FF4 | 541 (70.7%) | 335 (68.1%) | | |
| Incident (pre)diabetes at FF4 | 224 (29.3%) | 157 (31.9%) | OR | 1.13 |
| Male | 43.3% | 40.0% | OR.adj | 1.17 |
| Mean age at F4 | 50.7 | 50.2 | OR.adj | 1.17 |
| Mean BMI at F4 | 26.3 | 26.2 | OR.adj | 1.15 |
| Mean years of education | 12.1 | 12.1 | OR.adj | 1.14 |
| Ever smoker at F4 | 57.0% | 59.6% | OR.adj | 1.13 |
| Physically active at F4 | 61.7% | 60.4% | OR.adj | 1.13 |
| Parental diabetes | 21.0% | 21.3% | OR.adj | 1.13 |
| Hypertension (>140/90mmHg) at F4 | 10.8% | 9.8% | OR.adj | 1.14 |
| Mean triglycerides (mmol/l) at F4 | 1.17 | 1.15 | OR.adj | 1.15 |
| Mean cholesterol/HDL at F4 | 3.82 | 3.84 | OR.adj | 1.13 |
| Mean HOMA-IR at F4 | 1.85 | 1.87 | OR.adj | 1.13 |
| Mean fasting glucose (mmol/l) at F4 | 5.0 | 5.0 | OR.adj | 1.27 |
| All confounders combined | | | OR.adj | 1.26 |

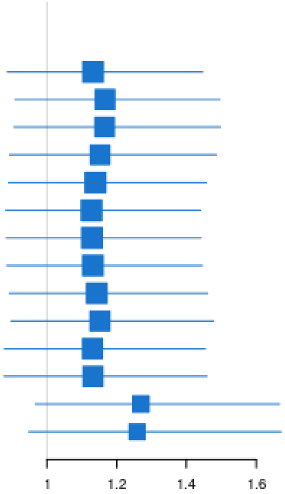


*ESM Figure 28: Contingency table and contribution of individual covariates for the association of HHV6 with (pre)diabetes incidence*

| At risk at F4 n=1257 | 182 HHV7- | 1075 HHV7+ | | |
|---|---|---|---|---|
| Normal glucose tolerance at FF4 | 120 (65.9%) | 756 (70.3%) | | |
| Incident (pre)diabetes at FF4 | 62 (34.1%) | 319 (29.7%) | OR | 0.82 |
| Male | 53.3% | 40.1% | OR.adj | 0.90 |
| Mean age at F4 | 51.4 | 50.4 | OR.adj | 0.85 |
| Mean BMI at F4 | 26.6 | 26.2 | OR.adj | 0.85 |
| Mean years of education | 12.4 | 12.1 | OR.adj | 0.79 |
| Ever smoker at F4 | 56.6% | 58.2% | OR.adj | 0.81 |
| Physically active at F4 | 58.8% | 61.6% | OR.adj | 0.82 |
| Parental diabetes | 18.1% | 21.7% | OR.adj | 0.81 |
| Hypertension (>140/90mmHg) at F4 | 8.2% | 10.8% | OR.adj | 0.80 |
| Mean triglycerides (mmol/l) at F4 | 1.18 | 1.16 | OR.adj | 0.82 |
| Mean cholesterol/HDL at F4 | 3.90 | 3.82 | OR.adj | 0.84 |
| Mean HOMA-IR at F4 | 1.91 | 1.85 | OR.adj | 0.84 |
| Mean fasting glucose (mmol/l) at F4 | 5.0 | 5.0 | OR.adj | 0.97 |
| All confounders combined | | | OR.adj | 0.99 |

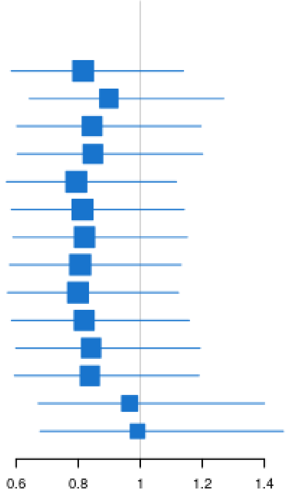


*ESM Figure 29: Contingency table and contribution of individual covariates for the association of HHV7 with (pre)diabetes incidence*

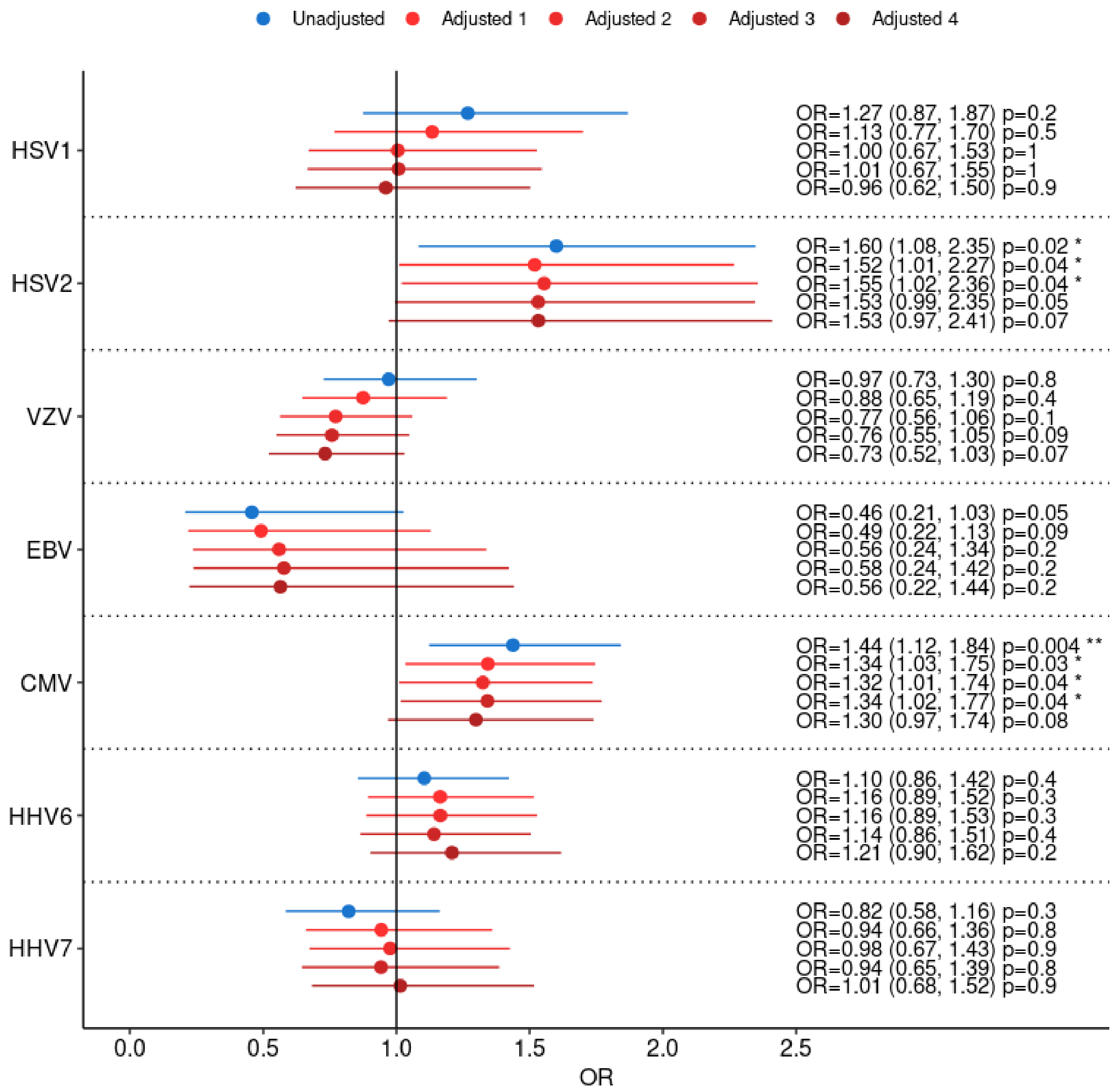


*ESM Figure 30: Mutually adjusted associations of herpesvirus seropositivity with incidence of (pre)diabetes (n=1257, 95% CI in brackets)*

Results are presented for unadjusted models and for models adjusted for:

1) sex, age

2) adjusted 1 plus BMI, education, smoking, physical activity

3) adjusted 2 plus parental diabetes, hypertension, triglycerides, total cholesterol/HDL, HOMA–IR

4) adjusted 3 plus fasting glucose

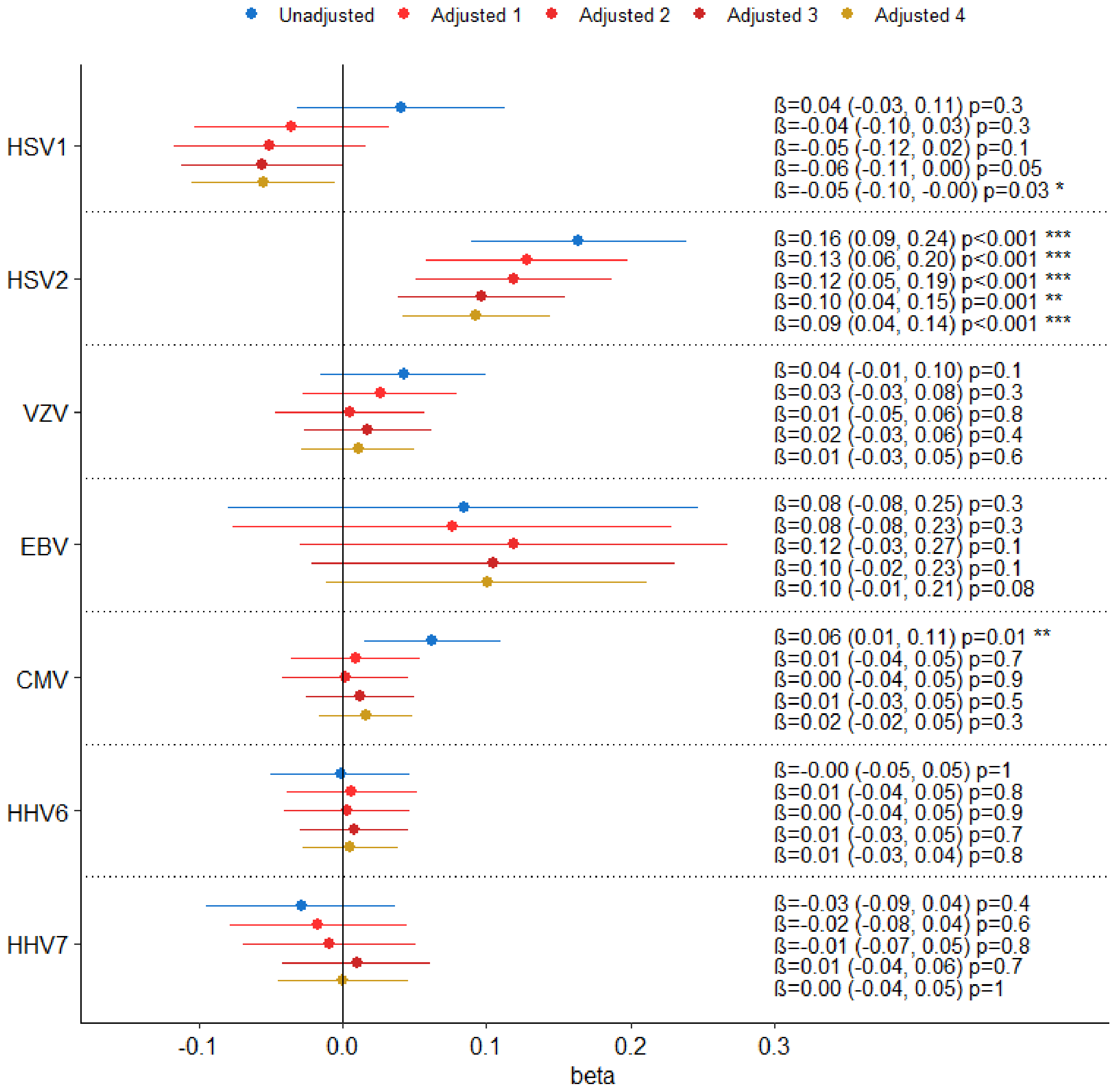


*ESM Figure 31: Mutually adjusted cross–sectional association of herpesvirus serostatus with HbA1c at baseline (n=1967, 95% CI in brackets)*

We report the regression coefficients (β and 95% CI) for unadjusted models and models adjusted for:

1) sex, age

2) adjusted 1 plus BMI, education, smoking, physical activity

3) adjusted 2 plus parental diabetes, hypertension, triglycerides, total cholesterol/HDL, HOMA–IR

4) adjusted 3 plus prevalence of (pre)diabetes
